# First-Principles Molecular Dynamics Simulations of Ammonia Adsorption onto MFI Zeolite Nanosheets

**DOI:** 10.1021/acsnanoscienceau.5c00151

**Published:** 2025-12-29

**Authors:** Ramanish Singh, Nathan Wang, Henry Wolters, Prerna, Michael Tsapatsis, J. Ilja Siepmann, Daniela Kohen

**Affiliations:** † Department of Chemistry and Chemical Theory Center, 5635 University of Minnesota, 207 Pleasant Street SE, Minneapolis, Minneapolis, Minnesota 55455, United States; ‡ Department of Chemical Engineering and Materials Science, University of Minnesota, 412 Washington Avenue SE, Minneapolis, Minnesota 55455, United States; § Chemistry Department, 6092 Carleton College, Northfield, Minnesota 55057, United States; ∥ Institute for NanoBioTechnology, 1466 Johns Hopkins University, Baltimore, Maryland 21218, United States; ⊥ Department of Chemical and Biomolecular Engineering, Johns Hopkins University, Baltimore, Maryland 21218, United States; # Applied Physics Laboratory, Johns Hopkins University, Laurel, Maryland 20723, United States

**Keywords:** ammonia, ammonium, silanol, zeolite, reactive adsorption, molecular dynamics

## Abstract

MFI zeolite nanosheet membranes are promising candidates for ammonia separation from nitrogen and hydrogen gases, yet questions remain on the origin of their high selectivity. Silanols, Si–OH, are present in high concentration at the surface of zeolite nanosheets, and force-field-based simulations indicate that surface adsorption at the silanols contributes to selectivity. Acidic silanol groups can chemically react with ammonia via transfer of a proton to form ammonium ions, which may further contribute to the ability of zeolite nanosheet membranes to separate ammonia from other gases. In this work, we used first-principles molecular dynamics techniques to simulate ammonia’s behavior within stacked MFI zeolite nanosheets. We found that at 523 K and a loading corresponding to 35 bar, conditions desired for the ammonia separation, about 30% of ammonia reacts with surface silanols. Our work explored H-bonding and proton transfer within this system.

## Graphical abstract

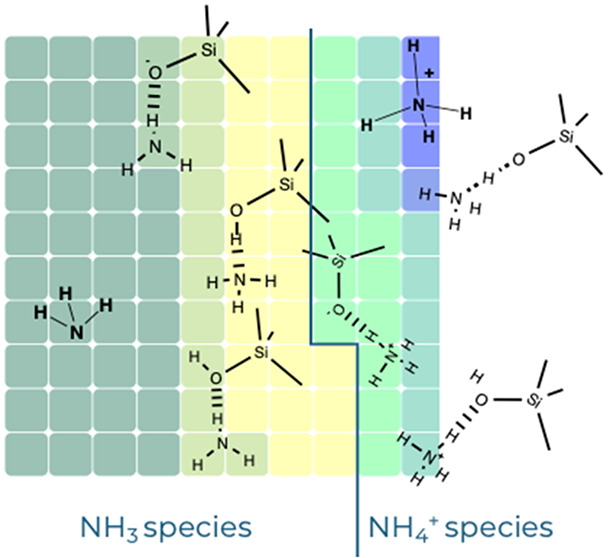

The fertilizer manufacturing process, crucial for food production, relies on ammonia (NH_3_) synthesis.^1^ Industrially, ammonia synthesis occurs at high temperatures (650–750 K) and high pressure (100–250 bar), following the protocol developed by Haber and Bosch more than a century ago,^2,3^ and is responsible for around 2% of the world’s total final energy consumption.^4^ Small-scale plants for ammonia production that rely on solar or wind energy can potentially mitigate this cost,^5^ but they face considerable economic challenges, including the separation of the product from the unreacted reactants, nitrogen (N_2_) and hydrogen (H_2_) gases. Separations under conditions closer to those of the catalytic reaction would reduce this cost, and membrane-based separation processes are a promising alternative to the current technology that relies on significant cooling to achieve NH_3_ condensation before that of H_2_ or N_2_.

Recently, Duan et al.^6^ demonstrated that MFI zeolite nanosheet membranes exhibit high fluxes and separation factors for binary NH_3_/H_2_ and NH_3_/N_2_ mixtures at *T* = 298 K and *P* = 3 bar. Silanols, Si–OH, are present in high concentrations at the surface of these zeolite nanosheets. Using non-reactive, force-field-based simulations, Patel et al.^7^ showed that an idealized MFI zeolite nanosheet may offer selectivity also at temperature–pressure conditions (*T* = 523 or 623 K and *P* = 80 bar) approaching those of the Haber-Bosch process. However, ammonia can chemically react with acidic silanols^8,9^ to form ammonium ions (NH_4_^+^), which may further contribute to the ability of zeolite nanosheet membranes to separate NH_3_ from H_2_ and N_2_ reactants. To gain insight into ammonia’s behavior within stacked MFI zeolite nanosheets with surface silanols and silanols bridging between nanosheets, we employed first-principles molecular dynamics (FPMD) simulations with interactions described by Kohn–Sham density functional theory, as this method is particularly well-suited for studying the breaking and forming of bonds that lead to the creation of ammonium (NH_4_^+^) ions.^10–14^ The model system (see Figure 1) consisting of 1 × 1.5 × 1 MFI unit cells was simulated at 523 K with a loading of 14 NH_3_ molecules reflecting adsorption at 35 bar, i.e., a state point that approaches ammonia synthesis conditions. Three independent simulation trajectories were carried out that differed in the initial NH_3_/NH_4_^+^ speciation and/or some simulation parameters, and these were used to obtain four segments (referred to as U0, U7, C0–1, and C0–2) for analysis (see Table 1 and Methods section).

**Figure 1:**
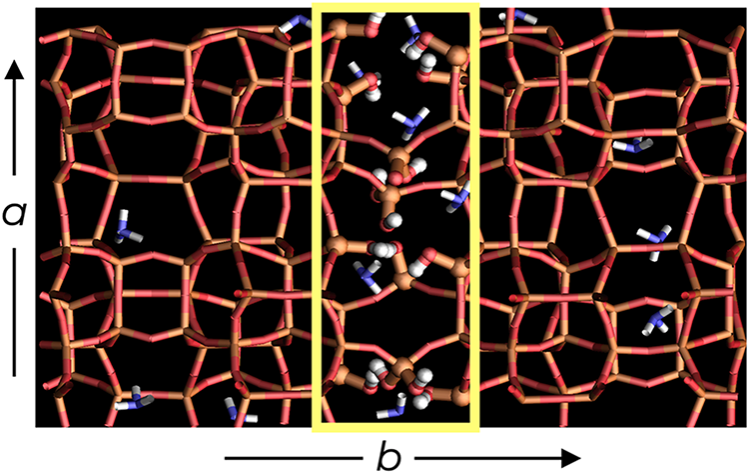
Initial configuration of the system used for trajectories U0 and C0 viewed along the *c*-axis. The intersheet gallery, enclosed in the yellow box in the picture, contains 4 (geminal) *q*_2_-silanols, Si­(OSi)_2_(OH)_2_, bridging between the sheets and 8 (vicinal) *q*_3_-silanols, Si­(OSi)_3_OH. All atoms in the simulation cell are shown; red corresponds to oxygen, gold to silicon, blue to nitrogen, and white to hydrogen atoms. Silanols and ammonia molecules are shown as spheres, and framework bonds as bars.

**Table 1:** Summary of Simulation Details Used for the Three Independent Simulation Trajectories and Four Production Segments

trajectory name	initial speciation	basis set/cutoff	total length [ps]	segment period [ps]
U0	14 NH_3_	MOLOPT-DZVP/600 Ry	90	50–90
U7	7 NH_3_ & 7 NH_4_^+^	MOLOPT-DZVP/600 Ry	90	50–90
C0	14 NH_3_	TZV2P/300 Ry	130	C0–1: 50–90
				C0–2: 90–130

## Results and Discussion

To evaluate the formation of ammonium ions, we need to find a criterion to distinguish covalent and hydrogen bonds. We accomplish this by computing radial distribution functions (RDFs), as they provide information about the preferred distances within a system. Figure 2 shows the RDFs, *g*(*r*_XH_) for N–H and O–H distances (where the latter considers only the silanol O atoms). Note that due to the heterogeneous nature of the nanosheet system, the RDFs only approach unity at distances larger than the size of the periodic cell. The first and second peaks in each type of RDF correspond to the most likely distances for the X-H covalent bond and for the X···H hydrogen bond (where X is the H-bond acceptor), respectively. We use the first and second minima as the upper bounds for covalent and H-bonds, respectively. Specifically, these bounds are 1.35 and 2.50 Å for covalent and H-bonds with N atoms, and 1.30 and 2.35 Å for covalent and H-bonds with O atoms. N atoms covalently bonded to three H atoms are classified as NH_3_, while those with four are classified as NH_4_^+^. The dashed lines in Figure 2 correspond to the number integrals, *n*(*r*_XH_), and represent the average number of H atoms surrounding the central X atom within a specific distance. The fact that *n*_NH_ at 1.35 Å is larger than 3 for all four simulation segments indicates that, on average, both NH_4_^+^ ions and NH_3_ molecules are present in significant fractions. Concomitantly, *n*_OH_ at 1.30 Å is less than unity, indicating that some of the silanols dissociate in the presence of ammonia.

**Figure 2:**
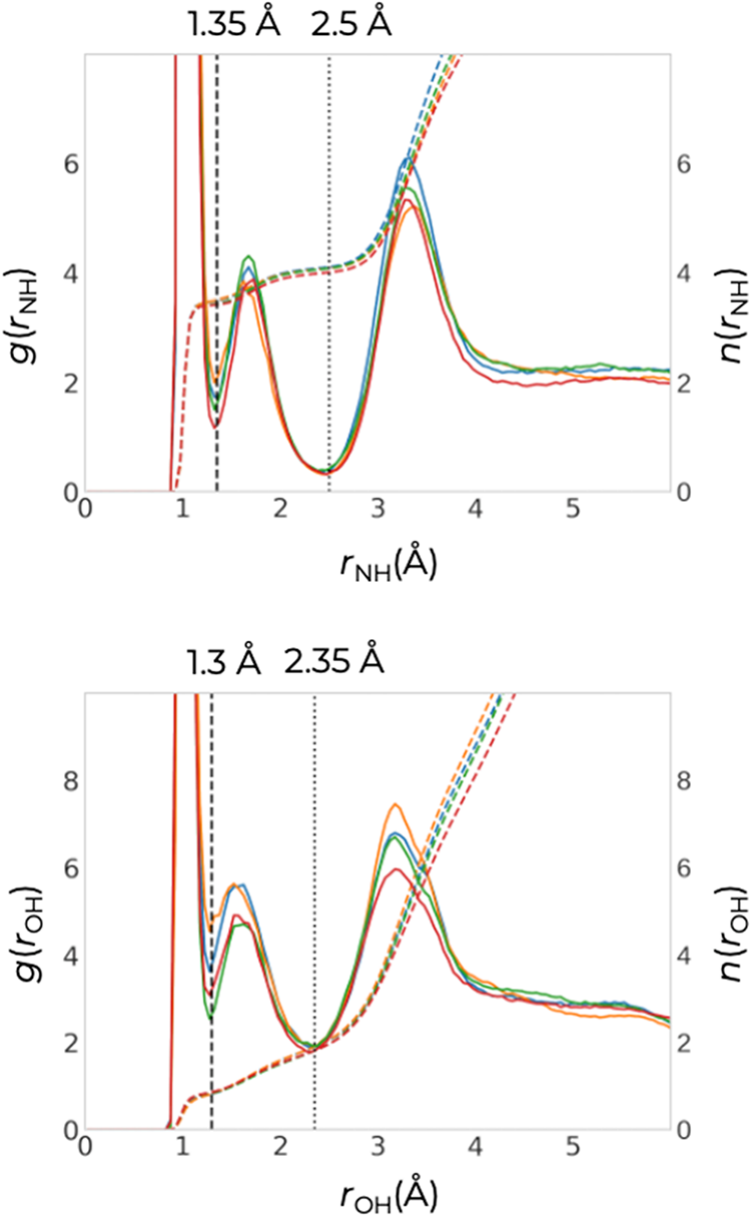
RDFs (solid lines) and their number integrals (dashed lines) for N–H (top) and O–H (bottom, only considering the silanol O atoms) pairs obtained from the U7 (blue), U0 (orange), C0–1 (green), and C0–2 (red) production segments. Cutoffs used for covalent and H-bonds are highlighted as vertical lines. The bin width for these analyses is 0.05 **Å**.

From Figure 3, we observe that 50 ps appears sufficient to allow the NH_3_/NH_4_^+^ speciation to equilibrate. Although the numbers fluctuate as should be the case for such small systems, about 30% of the N-containing species can be described as NH_4_^+^ during the production period of the simulations. It is encouraging to note that the U0 and U7 trajectories, started with different initial amounts of NH_4_^+^ ions, converge to nearly identical average values, while the simulations performed with different models (basis sets and plane wave cutoffs) produce somewhat different speciation. In the case of U7 and U0, the average is 4.6 and 4.7 NH_4_^+^ ions, respectively, out of a total of 14 N-containing species; while for C0–1 and C0–2, it is 3.8 and 3.7 NH_4_^+^ ions, respectively. Averaging over all four segments, we find 4.2 NH_4_^+^ ions or 30% of the N-containing species; i.e., a significant fraction of the ammonia molecules indeed reacts with surface silanols.

**Figure 3:**
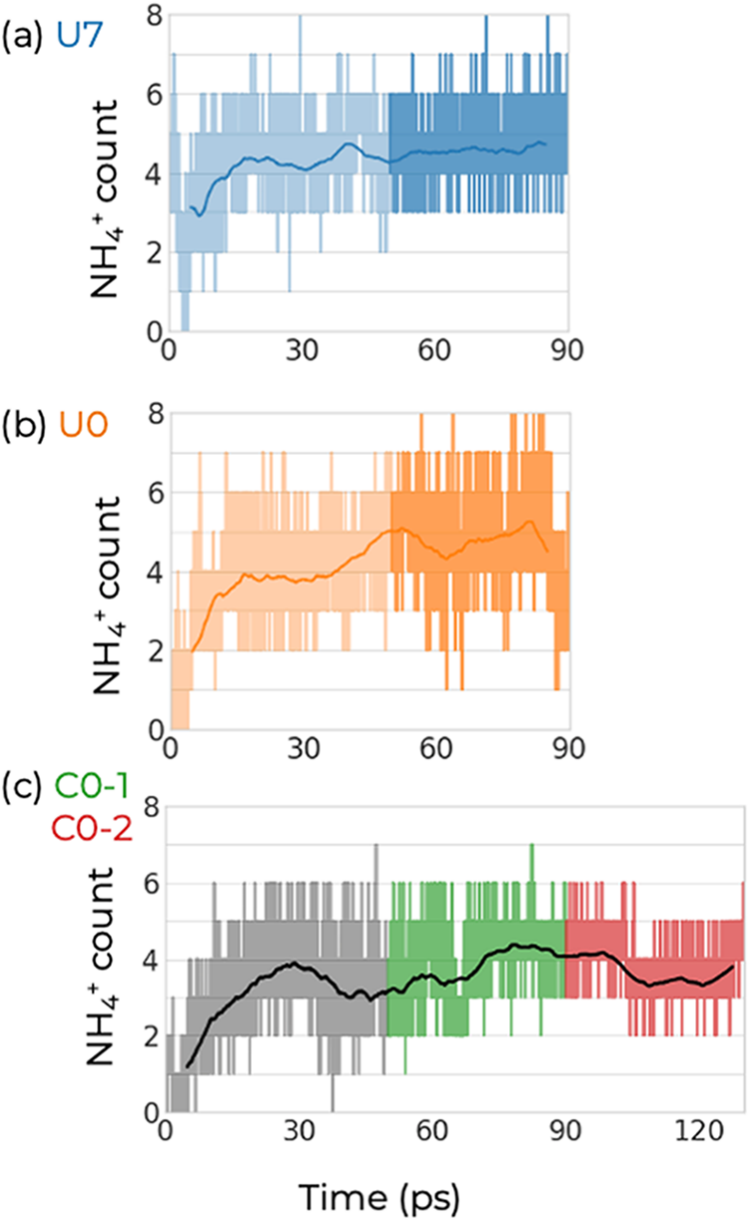
Number of ammonium ions present in trajectories (a) U7, (b) U0, and (c) C0. For the U7 and U0 trajectories, darker colors highlight the production portion, while for the C0 trajectory, the equilibration and C0–1, and C0–2 periods are shown in black, green, and red, respectively. A smoothed average (10 ps window) is shown as a bold line to guide the eye. The NH_3_/NH_4_^+^ speciation is determined using a 1.35 **Å** cutoff for N–H bonds. All systems contained 14 N atoms. Note how all three trajectories appear to have reached chemical equilibrium after 50 ps.

For the species identified as NH_4_^+^ to be a well-formed ammonium ion, the four hydrogen atoms should be arranged in a tetrahedral geometry. Figure S1 shows a histogram of the tetrahedral order parameter,^15^ and clearly illustrates that the bonding arrangement of the NH_4_^+^ ions is highly tetrahedral. Figure S2 shows the distributions for the shortest and longest N–H distances in NH_4_^+^ ions and NH_3_ molecules. For NH_4_^+^ ions, there is a significant shift in peak position (from 1.02 to 1.09 **Å**), tail of the distribution for the longest distances, and discontinuity at 1.35 Å, the upper bound used here for a covalent N–H bond. As will be discussed later, about 50% of the NH_4_^+^ ions participate as donors in an H-bond to a deprotonated Si–O^–^ species and 7% share a proton to form an H_3_NHO-Si complex. Thus, while maintaining a tetrahedral bonding geometry, the bond length distribution is asymmetric for the NH_4_^+^ ions. For the NH_3_ molecules, the distributions of the shortest and longest N–H distance exhibit a smaller shift (from 1.02 to 1.06 Å), and the distribution for the longest distance decays to zero at 1.20 Å.

We also expect that NH_4_^+^ ions will stay near the silanols in the intersheet gallery region (−3.75 Å ≤ *y* ≤ + 3.75 Å), while the ammonia molecules should be able to explore the interior of the zeolite nanosheet. Figure 4 shows that this is true for this system. Although there is some variation from simulation to simulation, note that in all cases, the NH_4_^+^ ions are consistently located near the silanols. In contrast, some NH_3_ molecules are found in the interior of the zeolite, particularly near ± 11 Å, the location of the sinusoidal channels (see Figure S3)

**Figure 4:**
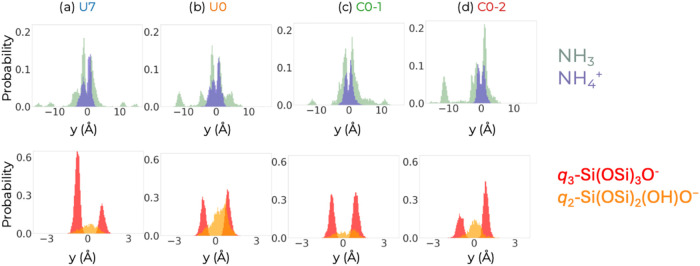
Histograms displaying positions of N-species (top) and deprotonated silanols (bottom) for the four production segments: (a) U7, (b) U0, (c) C0-1, and (d) C0-2. The bin size is 0.25 Å for the graphs on the top, and 0.05 Å for those on the bottom. The top distributions were normalized such that the area under both the NH_3_ and the NH_4_^+^ bars adds to one, while the normalization factor for the bottom graphs is such that the area under the silanol bars would sum to one if all are found as *q*_2_-Si­(OSi)_2_(OH)­O^–^ or if all are found as *q*_3_-Si­(OSi)_3_O^–^.

The covalent cutoff of *r*_OH_ = 1.30 Å was used to determine the protonation state of the silanols. From the area under the peaks in Figure 4 (bottom), it can be deduced that the *q*_2_-silanols belonging to the intersheet bridges are less reactive than the *q*_3_-silanols located on the two surfaces of the intersheet gallery. Specifically, averaging over the four production segments, about 16% of the *q*_2_-silanols have lost a single proton (their losing two protons is, as should be expected, extremely rare with a probability of less than 0.05%), whereas 35% of the *q*_3_-silanols have transferred a proton to form an NH_4_^+^ ion. The observation that the bridging *q*_2_-silanols are less reactive agrees with prior FPMD simulations of the amorphous silica/water interface by Pfeiffer-Laplaud et al.^14^ that found p*K*_a_ values of 8.9 and 2.1 for concave geminal and solvent-stabilized vicinal silanols.

To probe whether proton transfer leads to the formation of a static ion pair, we tracked the speciation of each of the 14 N atoms as a function of time during the 40 ps production segments (see Figure 5). Most N atoms switch between NH_3_ and NH_4_^+^, which indicates a relatively facile proton exchange for NH_*x*_ located in the intersheet gallery region. Some N atoms persist as an NH_3_ molecule through the entire 40 ps, but these are found in the interior region of the MFI nanosheet where they cannot react with silanol groups. Considering all four segments, only 2 N atoms in system U7 remain as NH_4_^+^ ions for more than 90% of the time. Together with the information from the spatial distribution of the N-species (Figure 4), we surmise that diffusion through the membrane in the *y*-direction (perpendicular to the sheets) will involve changes in speciation. An analysis of the square displacement for the N atoms in the *y*-direction shows that only one atom traveled the equivalent of the square of the *y*-dimension of the simulation box over the 130 ps of simulation trajectory C0, and a log–log plot of the mean-square displacement indicates that the diffusive regime has not been reached.

**Figure 5:**
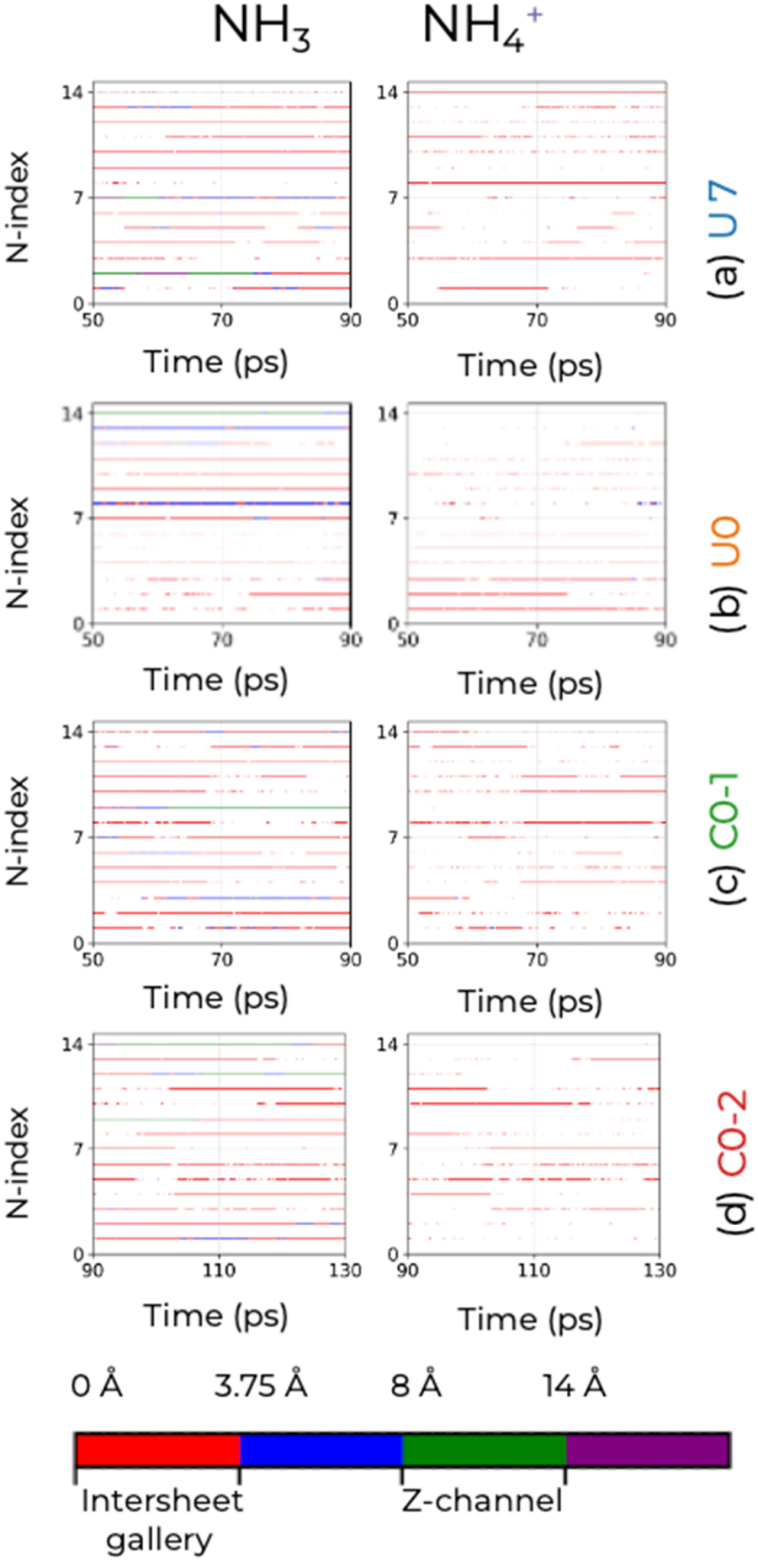
Identity of NH_*x*_ species as a function of time for the four production segments (a) U7, (b) U0, (c) C0–1, and (d) C0–2. The left and right columns correspond to the NH_3_ molecules and NH_4_^+^ ions, respectively. The distance from the center of the intersheet gallery region is denoted by the color of the points.

Combining both covalent and hydrogen bonding information, we can divide the NH_3+*y*_···H_1–*y*_OSi pairs into eight classes. Figure 6 and Table 2 present the abundances of the N-containing species. The majority of NH_*x*_ species interact strongly with the silanol species, but 39% of them are found as “free” NH_3_ molecules (i.e., not H-bonded to SiO_*x*_). About 18 and 10% of the NH_*x*_ species (corresponding to 26 and 14% of the NH_3_ molecules) are NH_3_ molecules accepting or donating a H-bond to a Si–OH group, respectively. An NH_3_ molecule acting as an H-bond donor to Si–O^–^ is relatively rare. For the NH_4_^+^ ions, the most prevalent class is being H-bonded to an Si–O^–^ group, but about one-third of NH_4_^+^ ions donate a H-bond to an Si–OH group. Using the covalent bond distance cutoffs, about 2% and 3% of the NH_*x*_ species are found in a proton-sharing arrangement (H_3_NHO, all bonds classified as covalent) or as “free” NH_4_^+^ ions, respectively (i.e., not involved in an H-bond, but still restricted by Coulomb interactions to the intersheet gallery).

**Figure 6:**
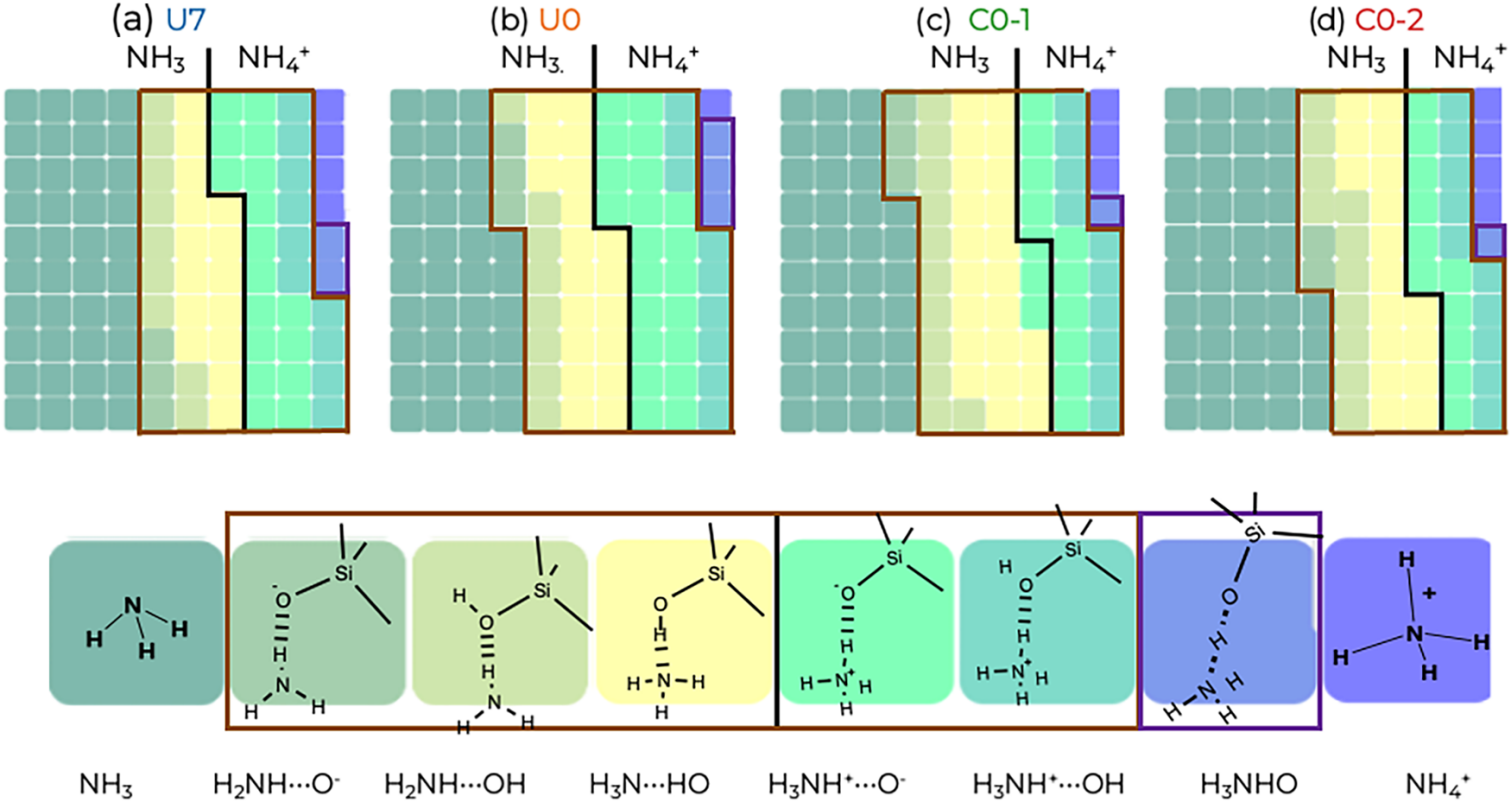
Waffle charts for each N-species obtained for the four production segments (a) U7, (b) U0, (c) C0–1, and (d) C0–2. Each square represents 1% of the total number of N-containing species (Table 2). A black line distinguishes all ammonia from ammonium squares; a brown box highlights squares indicating hydrogen-bonding to silanols, and a purple box surrounds the squares corresponding to H atoms shared between N-species and silanol. The color key at the bottom depicts cartoons of the eight classes.

**Table 2:** Abundance of N-Containing Species (in Percent) for the Four Production Segments and as an Unweighted Average over All Four Segments

species	U7	U0	C0–1	C0–2	average
NH_3_	40	36	37	44	39
H_2_NH···O^–^	3.3	3.3	3.0	2.1	3
H_2_NH···OH	9.1	8.2	11	11	10
H_3_N···HO	15	19	22	17	18
H_3_NH^+^···O^–^	17	21	13	11	15
H_3_NH^+^···OH	10	9	10	10	10
H_3_NHO	2.3	3.1	1.5	1.0	2
NH_4_^+^	3.9	0.9	2.6	4.4	3

To further characterize the structure of this complex system, we computed HN–HO pair pair distributions (PPDs). Figure 7 shows PPDs for all NH_*x*_ species, only the NH_3_ molecules, and only the NH_4_^+^ ions taken from the 80 ps combined production period of simulation C0. The corresponding PPDs for the U0 and U7 trajectories are presented in Figure S4. The combined PPD shows a curved region at small H–O and H–N distances with a saddle point in the region where the proton is classified as being shared with both the O and N atoms. There is a tiny region of enhanced probability (with a height between 1 and 4) just outside the two covalent bond cutoffs. However, shifting the distance cutoffs outward by 0.05 Å would not yield a significant increase in the fraction of proton-shared H_3_NHO species. The two peaks closest to the bottom left corner correspond to a silanol proton acting as a donor to form a H-bond to a neighboring N atom and to an NH_*x*_ proton acting as a donor to a neighboring O atom.

**Figure 7:**
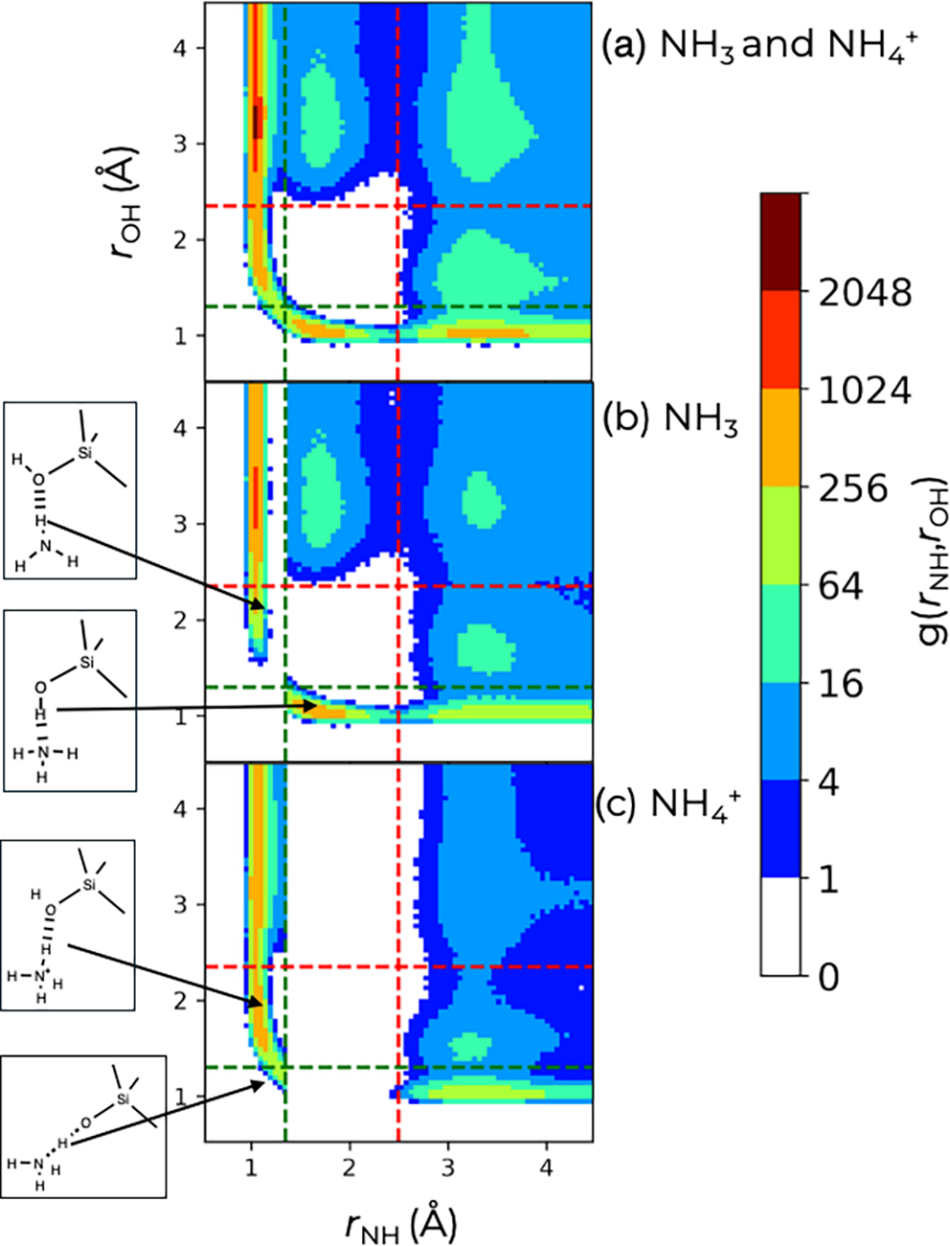
Heat maps for the HN–HO pair–pair distributions calculated for the C0 trajectory from 50 to 130 ps. (a, top) considers all NH_*x*_ species, while (b, middle) focus on NH_3_ molecules and (c, bottom) on NH_4_^+^ ions. Green and red dashed lines delineate the covalent and hydrogen bond cutoffs.

When the PPDs are analyzed separately for only the NH_3_ molecules and for only NH_4_^+^ ions, the continuous curved region gets broken. The low-probability region at 1.2 Å ≤ *r*_HN_ ≤ 1.35 Å for all *r*_HO_, in the PPD corresponding to the NH_3_ molecules, indicates that the distribution of covalent bond length is quite sharp in this case (also see Figure S2). When an NH_3_ molecule is the H-bond donor, then the distance to the O acceptor is always larger than 1.5 Å. In contrast, when the NH_4_^+^ ion acts as the donor, then the length of the H-bond to the O acceptor extends to shorter distances, but concomitantly involves a lengthening of the covalent H–N bond. Of course, only the NH_4_^+^ ions contribute to the proton-shared region. Focusing on the region corresponding to H covalently bonded to a silanol oxygen, there is only a very low probability in the region of 1.35 Å ≤ *r*_HN_ ≤ 2.5 Å because the absence of a lone electron pair on the NH_4_^+^ ion does not allow for the formation of a H-bond with the ion as an acceptor.

## Conclusions

FPMD simulations were conducted to investigate how ammonia interacts with stacked MFI zeolite nanosheets, as membranes formed by them show promise for energy-efficient ammonia purification during its synthesis. Simulations performed at 523 K indicate that about 30% of NH_3_ molecules react with surface silanols to form NH_4_^+^ ions. Due to cation–anion attractions, the NH_4_^+^ ions remain in the intersheet gallery region with the corresponding number of deprotonated silanols, while NH_3_ can also explore the interior region of the nanosheets. However, even during the relatively short FPMD simulations, frequent changes in the NH_3_/NH_4_^+^ speciation are observed, while the diffusive regime is not reached. At the conditions investigated here, about 60% of the nitrogen-containing species are H-bonded to silanols, with NH_4_^+^ forming stronger H-bonds or binding covalently to a silanol O atom through a shared proton. Our FPMD simulations indicate that surface silanols play a crucial role in the selective adsorption of ammonia in MFI zeolite membranes and the separation from N_2_ and H_2_ under synthesis-relevant conditions.

## Methods

The system contains an MFI zeolite nanosheet consisting of 1 × 1.5 × 1 unit cells that is periodically replicated in all three directions. The straight channel of the MFI framework aligned with the crystallographic *b* axis runs perpendicular through the nanosheet with the [010] surface exposed and terminated by eight *q*_3_-SiOH groups (i.e., their Si atom is connected to three other Si atoms through an O atom) per surface. The all-silica zeolite framework MFI/silicalite-1 coordinates used were those obtained by van Koningsveld et al.^16^ To mimic a secondary zeolite growth, four SiO_2_ units were added in the gallery space between the two [010] surfaces, and the Feuston-Garofalini potential^17^ was used to allow these units to react and form *q*_2_-Si bridges with two silanol groups while interior Si and O atoms were kept at their crystallographic locations. The periodic box used for the simulations has dimensions of *a* = 20.021963 Å, *b* = 32.17182 Å, and *c* = 13.38965 Å and contains 148 Si, 304 O, and 16 H atoms.

Force-field-based simulations in the Gibbs ensemble using the simulation parameters described by Patel et al.^7^ but with a short cutoff at half the c-dimension (*r*_cut_ = 6.69 Å) were used to estimate the NH_3_ loading at 523 K and 30 to 80 bar. During these simulations, the NH_3_ molecules were treated as rigid (allowing only for translations, rotations, and transfers between reservoir and zeolite phases), and only the terminal −O-H groups of the *q*_3_ and *q*_2_-silanols were allowed to undergo angle bending and dihedral rotation, while all of the other framework Si and O atoms were kept frozen. The Gibbs ensemble simulations yielded a loading close to 14 NH_3_ molecules for the MFI nanosheet consisting of 1.5 unit cells (26.4 mg/g) at a pressure of 35 bar, which is significantly higher than the loading in a bulk MFI unit cell at this pressure due to the silanols being stronger adsorption sites. At 80 bar, the loading reached 20 NH_3_ molecules for the MFI nanosheet. Here, we decided to carry out the FPMD simulations for the system with 14 adsorbed NH_3_ molecules, which in total contains 148 Si, 304 O, 14 N, and 58 H atoms.

For this work, three different trajectories were run and analyzed. The initial configuration for runs U0 and C0 was taken directly from the force-field-based simulations and contained 14 NH_3_ molecules and no NH_4_^+^ ions (see Figure 1). Trajectories U0 and C0 differ with regard to the basis set and thermostat used. Since it was a priori not known whether acid–base reactions (proton transfers) between Si–OH groups and NH_3_ molecules would be facile on the time scale accessible to FPMD simulations, trajectory U7 was started with 7 NH_3_ molecules and 7 NH_4_^+^ ions. To this extent, the 7 N atoms closest to the H atom of a Si–OH group were selected, and the H atom was manually transferred to yield tetrahedrally coordinated NH_4_^+^ ions.

FPMD simulations were conducted using the CP2K simulation package,^18^ which employs Kohn–Sham density functional theory (DFT) to calculate forces acting on the nuclei “on the fly.” Its QUICKSTEP module utilizes the Gaussian and Plane Wave (GPW) method,^19^ where the electronic density is expanded based on a plane wave basis. The Perdew–Burke–Ernzerhof (PBE) functional^20^ and D3 dispersion correction from Grimme^21^ were applied. Core electrons were modeled with Goedecker–Teter–Hutter pseudopotentials.^22^

Nuclear motion was integrated using Newton’s equations with a time step of 0.5 fs. At each step, the wave function was optimized with the self-consistent field convergence criterion set to 10^–6^ a.u. Simulations were performed in the canonical (*NVT*) ensemble at *T* = 523 K.

To assess the robustness of the system’s behavior, minor variations in electronic structure and runtime parameters were employed. For trajectory C0, the TZV2P basis set^23^ and the CSVR thermostat, with a time constant of 100 fs, were used; while for trajectories U0 and U7, the MOLOPT-DZVP basis set^23^ and the Nosé-Hoover^24^ thermostat,^25^ with a time constant of 1000 fs, were used. For trajectory C0, the energy cutoff of the auxiliary plane wave basis was set to 300 Ry, while in trajectories U0 and U7, it was set to 600 Ry.

Before the start of the FPMD simulations, the initial configurations underwent a quick geometry optimization to reduce strain due to switching from the force field to the DFT representation. For trajectory C0, a two-step optimization procedure was used, consisting of a first optimization of the MFI nanosheet in the absence of ammonia, followed by a second optimization after the addition of 14 ammonia molecules; for these calculations, the energy cutoff of the auxiliary plane wave basis was set to 3000 Ry. A one-step optimization was used for trajectories U0 and U7. Files with initial coordinates for the MD simulations are included in the SI (Tables S1–S3).

To determine the length of the equilibration period, the NH_4_^+^ count (Figure 3) and kinetic temperature (Figure S5) were monitored. All three trajectories appear to have stabilized after 50 ps, as indicated by the NH_4_^+^ count (chemical equilibrium) and the temperature (thermal equilibrium) fluctuating around their mean values. The U0 and U7 trajectories were run for a total of 90 ps, while the C0 trajectory was run for 130 ps. Thus, four production segments consisting each of 40 ps were analyzed for the data presented in this work (the final 40 ps for the U0 and U7 trajectories, and the periods from 50 to 90 ps (C0–1) and from 90 to 130 ps (C0–2).

Radial (pair) distribution functions (RDFs) were computed by using a bin size of 0.05 Å. The minima reported are those of the average of the four production segments. Pair–pair distributions (PPDs) were computed using a straightforward extension of the RDFs as described in the Supporting Information.

## Supplementary Material





## Notes

Initial configurations for the three FPMD trajectories as xyz-files (ZIP)
Calculation details for RDFs and PPDs; supporting figures showing histograms of tetrahedral order parameter for NH_4_^+^ ions, N–H bond length distributions, traces of atomic positions; PPDs for the U0 and U7 trajectories; evolution of the temperature for the three simulations; and tables reporting the *xyz* coordinates after DFT optimization used as the initial configurations for the three FPMD trajectories (PDF)
