# Supplementary material for: First-Principles Molecular Dynamics Simulations of Ammonia Adsorption onto MFI Zeolite Nanosheets
Source: ACS Nanosci Au. 2025 Dec 29;6(2):263–9. doi: 10.1021/acsnanoscienceau.5c00151 (PMC13087932; doi:10.1021/acsnanoscienceau.5c00151)
Supplement: Supplementary file 2 [file ng5c00151_si_002.pdf]

Supplemental Information

First Principles Molecular Dynamics Simulations of Ammonia Adsorption onto MFI Zeolite Nanosheets

Ramanish Singh,[a,b] Nathan Wang,[c] Henry Wolters,[c] Prerna,[a,b]

Michael Tsapatsis,[d,e,f] J. Ilja Siepmann,[a,b] and Daniela Kohen[c]

[a] Department of Chemistry and Chemical Theory Center, University of Minnesota,

207 Pleasant Street SE, Minneapolis, Minneapolis, Minnesota 55455-0431, United States

[b] Department of Chemical Engineering and Materials Science, University of Minnesota,

412 Washington Avenue SE, Minneapolis, Minnesota 55455-0132, United States

[c] Chemistry Department, Carleton College, Northfield, MN

[d] Institute for NanoBioTechnology, Johns Hopkins University, Baltimore,

Maryland 21218, United States

[e] Department of Chemical and Biomolecular Engineering, Johns Hopkins University, Baltimore,

Maryland 21218, United States

[f] Applied Physics Laboratory, Johns Hopkins University, Laurel, Maryland 20723, United States

Radial Distribution Function and Pair−Pair Distribution

A radial distribution function (RDF) or $g(r)$ between pairs of atoms can be calculated using [Allen *et al.*, 1994]

$$g\left(r+\frac{1}{2}\delta r\right)=\frac{n(b)}{n^{id}(b)},\ \text{were}\ n^{id}(b)=\frac{N}{V}\frac{4\pi}{3}[(r+\delta r)^3-r^3]\ \text{and}\ n(b)=\frac{n_{his}(b)}{N\#conf}.$$

Therefore

$$g\left(r_{HX}+\frac{1}{2}\delta r_{HX}\right)=\frac{n_{his}(b)}{N_X N_H \#conf\frac{4\pi}{3}}\frac{V_{cell}}{[(r_{HX}+\delta r_{HX})^3-r_{HX}{}^3]}$$

By extension, the pair−pair distribution (PPD) was computed as:

$$\mathrm{g}\left(r_{HO}+\frac{1}{2}\delta r_{HO},r_{HN}+\frac{1}{2}\delta r_{HN}\right)=\frac{n_{his}(a,b)}{N_O N_N N_H \#conf\{(r_{HO}+\delta r_{HO})^3-r_{HO}{}^3\}\{(r_{HN}+\delta r_{HN})^3-r_{HN}{}^3\}}\left\{\frac{V_{cell}}{\frac{4\pi}{3}}\right\}^2$$

To obtain this plot, each H atom is considered one at a time, and the distances for that particular H atom to all silanols' O atoms and to all N atoms are computed and then binned. The validity of this approach was confirmed by demonstrating that the PPD for an ideal gas mixture consisting of 16 O, 14 N, and 58 H atoms is unity, bare noise, at all points.

Supplementary Figures

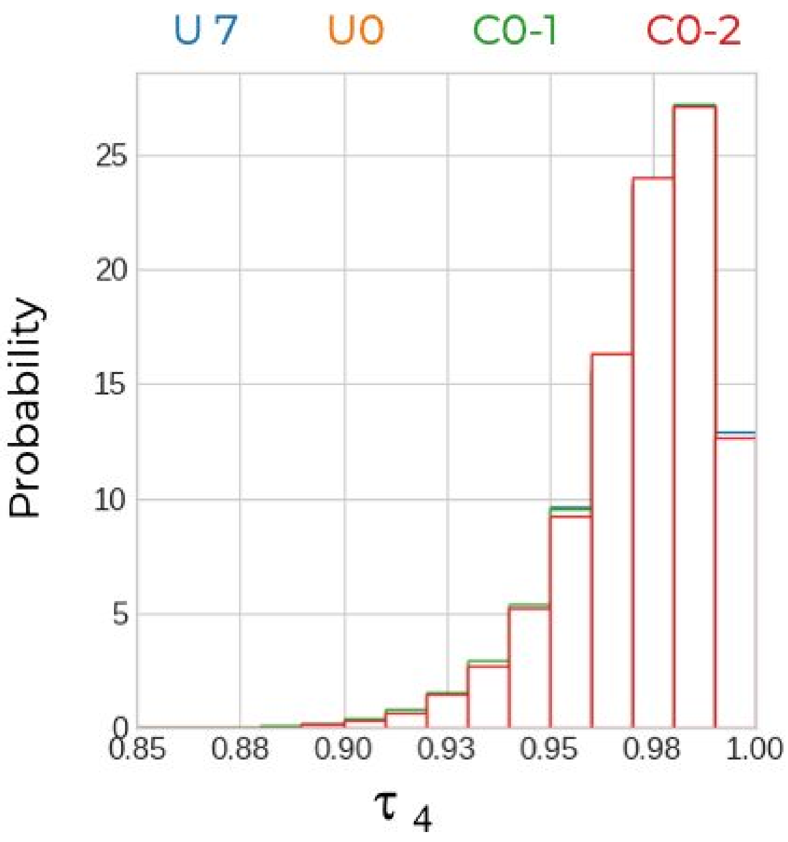

Figure S1. Histograms of the tetrahedral order parameter, τ_4, for the production segments U7 (blue), U0 (orange), C0-1 (green) and C0-2 (red).

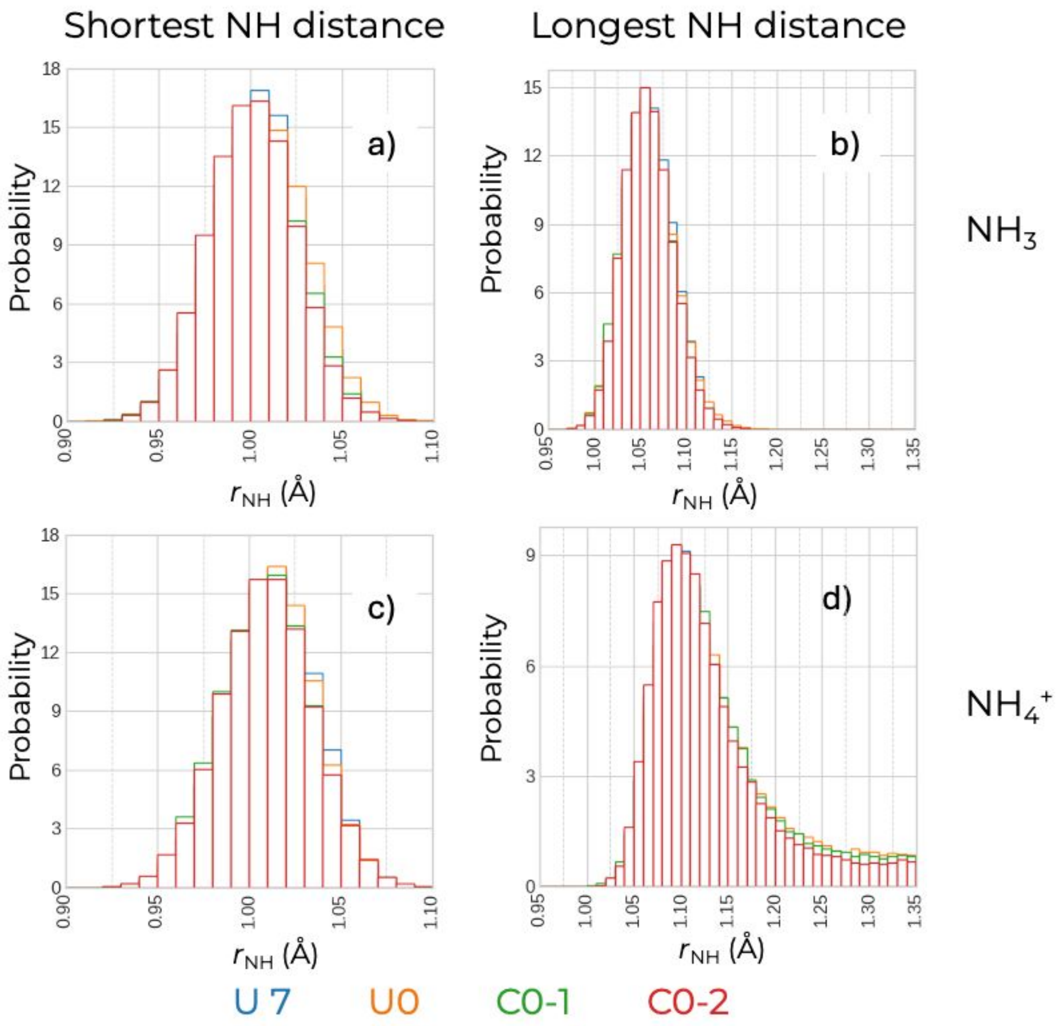

Figure S2. Histogram for the shortest (a and c) and longest (b and d) N-H distance in NH_3 molecules (a and b) and NH_4^+ ions (c and d). In all cases, the bin width is 0.01 Å.

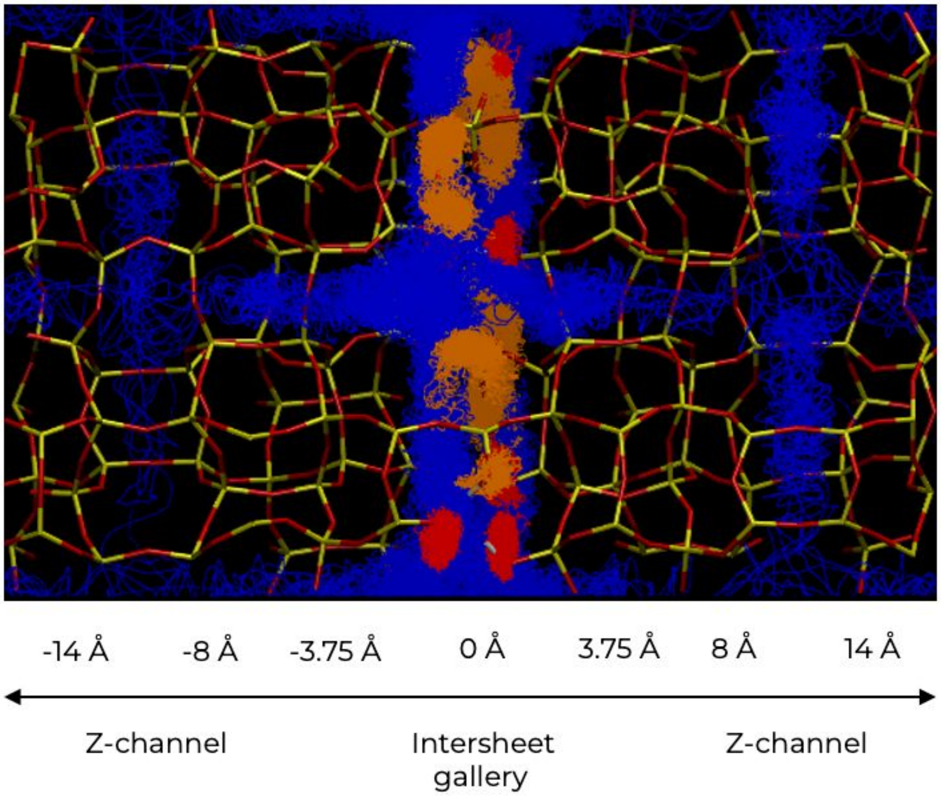

Figure S3. Atomic positions viewed along the *c*-axis obtained from the C0 trajectory. Blue, red, and orange traces correspond to N atoms of NH_x species, O atoms in q_3-silanol groups, and O atoms in q_2-silanol groups, respectively. Other Si and O framework atoms are depicted as yellow and red tubes, respectively.

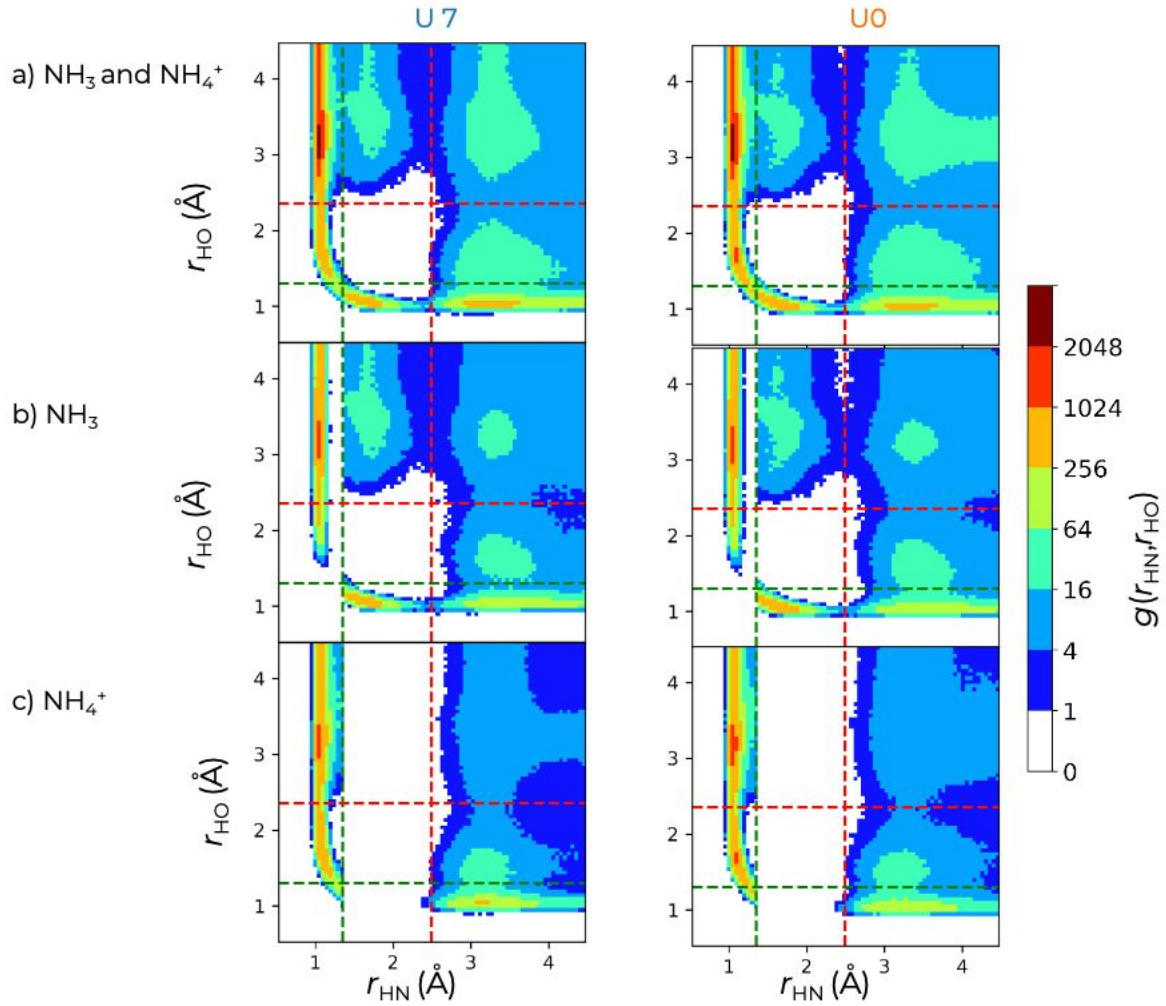

Figure S4. Pair−pair distributions for (a, top row) all N-species, (b, middle row) only for ammonia molecules, and (c, bottom row) only for ammonium ions obtained from the U7 (left) and U0 (right) trajectories.

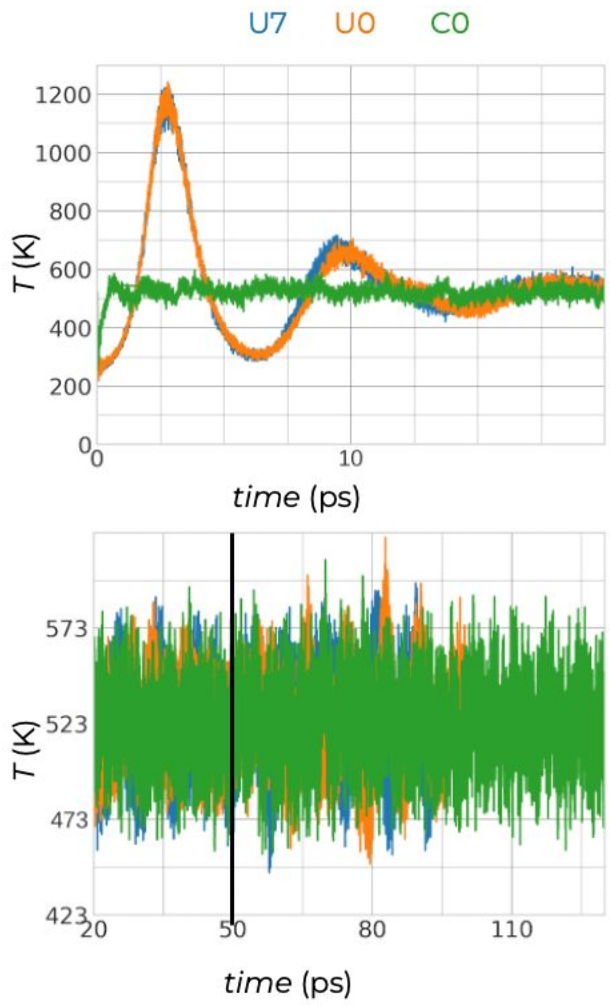

Figure S5. Kinetic temperature as a function of time: initial 20-ps periods (top) and remainder (bottom) of the three trajectories. Note that each trajectory has reached thermal equilibrium when the production period is started (50ps, black vertical line). The U7 and U0 trajectories used a thermostat with a longer time constant.

Table S1. Initial coordinates for the C0 MD simulation.

```
524
C0.INI.xyz
 Si        6.4519239409        3.3873138019       10.9174290842
 Si        1.5433473248        3.7389795792       11.0006385971
 Si        1.5615652593        5.1412655743        2.1964907821
 Si        3.9013524771        5.7317979102        4.1124195736
 Si        4.4188683597        4.9688186991        7.1883459615
 Si        7.5985752545        5.0041768941        7.0523723103
 Si        8.5933300243        5.3103448876        4.1085805587
 Si        6.2831875286        5.0140294176        2.1948257405
 Si       11.6405767414        4.8720970261        4.5486878058
 Si       13.9157867102        5.6540691592        2.5597437153
 Si       14.4823718618        4.7070604646       13.0829438257
 Si       17.6170310012        4.7480199200       13.0338020562
 Si       18.4592011442        5.3245645357        2.5747882191
 Si       16.2846156611        5.0424409864        4.6481914396
 Si       16.1203519852        3.2903722188        9.1901787802
 Si       11.5382483412        3.6777617444        9.0382628598
 Si        8.3942498384        7.3805759384        8.8564573786
 Si        6.2237717897        6.5271647371       11.0715423364
 Si        5.6177301902        7.5214095625        0.5044126233
 Si        2.4613525024        7.5597533424        0.3685681028
 Si        1.4644505329        6.9101889050       10.9233095136
 Si        3.8227068841        7.2905596031        9.1501601992
 Si        8.5262336690       22.7229556034        9.3545935982
 Si        6.0950437244       23.4159163284       11.1083735040
 Si        5.3722852634       22.5032210532        0.5740014400
 Si        2.2757992028       22.7389847314        0.6412632375
 Si        1.4240274247       23.6849373924       11.1503243902
 Si        3.7131918337       22.7265828026        9.1344172545
 Si        1.2752916623       25.1192883478        2.2679817617
 Si        3.8448271876       25.5511394363        4.0971063095
 Si        4.2803161429       24.8387242341        7.0682555857
 Si        7.4997974758       24.7738945243        7.2175375648
 Si        8.5436192692       25.2731621407        4.3772514970
 Si        6.2744192841       24.8296235206        2.3455059128
 Si        1.5144430791        9.6141065540        2.3816781277
 Si        3.8380678448        8.9179412265        4.3091854578
 Si        4.5745601393        9.6217701081        7.1635862842
 Si        7.6731032485        9.6497389597        6.9482699936
 Si        8.7145082697        8.5337449102        4.1418759665
 Si        6.2370034804        9.7806351140        2.5236474490
 Si       11.4781810765       17.4026684821        4.4376518870
 Si       13.7019260132       16.5679158254        2.3760204407
 Si       14.3789357169       17.0989298774       12.8710152740
 Si       17.4924068597       17.2564880967       12.9239026916
 Si       18.6053597380       16.8659608245        2.3385451785
 Si       16.1876030525       17.3696128623        4.0332180377
 Si       11.5495253500       12.6700589643        4.2758843182
 Si       13.7190064826       13.3849615410        2.2549665427
 Si       14.5541778079       12.6163069367       12.7423265402
```

Si	17.6145851497	12.5765393506	13.0166611085
Si	18.5784594260	13.7065525125	2.4421282837
Si	16.1818269735	12.7184333271	4.1222508047
Si	18.3181491143	14.7487515602	11.1064831170
Si	16.0642870555	15.4180120037	9.0852232558
Si	15.5639483440	15.0581319528	6.0901164364
Si	12.4190751553	14.9764464638	6.2418451063
Si	11.3264624265	15.4754187288	9.0886456601
Si	13.6784936075	14.7351483221	10.7835421254
Si	18.2348762630	19.4737912831	10.7517428883
Si	15.9794667166	18.5443417880	8.9715454377
Si	15.2996837434	19.4450285813	6.1470746349
Si	12.1019422327	19.7778423954	6.3433374931
Si	11.2042410475	18.6115414640	9.1561031826
Si	13.6200777893	19.4434422913	10.8646441549
Si	11.5628740084	25.1969083739	4.5037162153
Si	13.8588018578	25.7401995498	2.3988998096
Si	14.2911160627	24.9190546622	12.8634781825
Si	17.4415633841	24.7288278218	13.0976354506
Si	18.3446951020	25.5976308067	2.5023889649
Si	15.9691416319	24.7764305283	4.3125792426
Si	11.5448271896	9.5845225432	4.4861119111
Si	13.9504784082	8.7665682191	2.5010494672
Si	14.4937424334	9.5096449147	13.0177765859
Si	17.5440136991	9.4092416335	13.0519232981
Si	18.6379457168	8.5244728951	2.5498350588
Si	16.4010784848	9.5724704035	4.3409030486
Si	18.4824209335	7.1321265809	11.2292336429
Si	15.9712315135	6.4785895690	9.3298362100
Si	15.6534697887	7.4405030122	6.3827341593
Si	12.4757450016	7.3417316328	6.3699628659
Si	11.3290270704	6.7554435290	9.1937296612
Si	13.5290328525	7.1052654279	11.3453902673
Si	18.4334443167	22.5832578076	11.1846276305
Si	16.2918129143	23.4538334501	9.1889243935
Si	15.5870192039	22.5276620347	6.3197520929
Si	12.3937880027	22.8084651840	6.3262824196
Si	11.4695468462	23.7808061564	9.2268702953
Si	13.8700706719	22.5076692761	10.9584311991
Si	8.4450717032	15.0970880548	8.4968020702
Si	6.1183194940	15.6205778454	10.5268559758
Si	5.5409016466	14.8691469223	0.0296022171
Si	2.3436988078	14.7562115739	0.1129066359
Si	1.1963684279	15.4981415567	10.7415141398
Si	3.5487459645	15.1286867181	8.7988938468
Si	8.3637031070	19.6396575433	9.0806690084
Si	6.1211171753	18.7297836592	10.9964824056
Si	5.3342010686	19.5509150030	0.5171915469
Si	2.1353991531	19.7355141267	0.1484364596
Si	1.2027631495	18.6461548397	10.7252838354
Si	3.6919802136	19.5027674431	9.1354185129
Si	1.5275378892	17.2244508786	1.9674168754
Si	3.8226469737	16.5766130117	4.0629666539
Si	4.4169611891	17.4533888483	6.9057310733

Si	7.5701381674	17.6218770465	6.8756885333
Si	8.5482279037	16.8866842511	4.0709033913
Si	6.1847128039	17.0730592149	2.1435325014
Si	1.4745681275	12.7093488517	2.2959671557
Si	3.7938285986	13.4870019108	4.2545212976
Si	4.5245012425	12.5931896680	7.2147030205
Si	7.6853052141	12.6436072665	6.8017920060
Si	8.5524129497	13.7676300979	4.0694800311
Si	6.2913433637	12.9284430372	2.2735909997
Si	8.2473073773	27.2447530005	9.1330688775
Si	5.9357734190	26.5483456123	11.2358911074
Si	5.4398554276	27.3930811380	0.7750296434
Si	2.3093727584	27.3479919111	0.4400425955
Si	1.2660345506	26.8929701154	10.9449202046
Si	3.6450435732	27.1652145470	9.2015437789
Si	3.8325811873	28.6051303397	4.4944366652
Si	8.5232193145	28.4329602766	4.3928499569
Si	13.9379152538	28.8342180201	2.5100833052
Si	18.4854127873	28.6625141339	2.4097460588
Si	18.2809329582	27.1840500700	11.3598114773
Si	16.0472776094	26.6062468850	9.2111565998
Si	15.2371024689	27.1086212598	6.3513520487
Si	12.1171430661	27.4803809422	6.6129617131
Si	11.2227175188	26.9226306399	9.4960035910
Si	13.7052017550	27.3794099078	11.0365167220
Si	3.9151160590	2.6559902312	9.0672684547
Si	18.7198021103	2.7265075774	10.9908321153
Si	6.1087855107	29.6811692961	2.7832947959
Si	16.1910008435	29.5139207101	4.4247347809
Si	5.5979561078	2.5803748715	0.2431487230
Si	15.6619652927	2.3217675707	6.2240060103
Si	4.4674756359	29.4274648028	7.3135377309
Si	17.5386998438	29.7782189624	13.0667541291
O	6.1296762276	4.9213646182	11.3851040079
O	1.8382870527	5.3348155948	11.1948155265
O	8.3843927426	6.3192722626	7.6082043434
O	2.3983165475	5.4124614722	3.5723651729
O	3.9941753600	5.2058288065	5.6469000983
O	6.0208110413	5.1636362015	7.4484522940
O	7.7310976399	4.9274695357	5.4380186339
O	7.7393648876	4.6717373975	2.8668830956
O	5.0546341328	4.9203746968	3.2797235119
O	6.3255861212	6.5178307128	1.5792071938
O	10.0751603383	4.6132957185	4.1246208303
O	12.4693099688	5.2138716644	3.1802658807
O	14.2906075138	4.6699630733	1.3111621624
O	16.0412916313	4.8771699204	12.5989243201
O	17.7187002552	4.6940864698	1.2627891547
O	17.6937120105	4.6986761314	3.8853027176
O	15.1317180869	5.5497352372	3.6213533353
O	16.5616538024	6.2640378096	5.7017754801
O	0.0029082982	4.8696174141	2.6032729865
O	15.8650983381	4.8941152020	8.9354170371
O	10.9460986733	5.1943675705	8.8918717825

O	18.4207364325	6.0595138762	12.4715055002
O	7.4995719767	6.8450468775	10.1055656971
O	6.3867869764	7.3901629516	12.4550831557
O	4.0379269600	7.1208555859	0.2947865534
O	1.7772338929	7.7440668676	12.2922041262
O	2.3278221416	7.5973488036	9.7191747613
O	4.8018948208	6.9284399994	10.3916971314
O	7.5659056832	22.8212413988	10.6873488081
O	5.4844752086	22.5277409373	12.3299511163
O	3.8442006397	22.7474421381	1.0850176154
O	2.2132708465	23.4391380827	12.5569822379
O	2.3406023976	23.1188004426	9.9246840598
O	5.0478134498	23.3269361508	9.8674485235
O	6.2611012518	24.9781071783	11.5780931416
O	1.1554813079	25.2727567591	10.9620959963
O	7.8307675061	8.8222277558	8.3422125656
O	8.0737928301	26.1101183585	7.9662397935
O	7.7423736790	23.4456919946	8.1232139485
O	4.4635623993	8.5737141897	8.3912749471
O	3.6298088113	6.0230748986	8.1362040068
O	3.7245875662	23.4110905133	7.6483109427
O	19.9253278332	7.0445898831	10.4823355737
O	20.0260116038	22.9033855796	11.1697595274
O	2.4057198252	25.5153299382	3.3547191761
O	3.6061235964	24.9640825947	5.5937886270
O	5.9043048890	24.9231501860	6.9207691351
O	8.3171363325	24.5444781897	5.8252765335
O	7.5964267874	24.5917680849	3.2516002552
O	4.9414908699	24.6544181159	3.2871332314
O	2.4606139193	9.5210659437	3.6995959656
O	3.6541296106	9.1099130291	5.9189110964
O	6.0916924450	9.7212464604	6.5604896556
O	8.4534600609	8.9332961967	5.7078150624
O	7.6204303909	9.1565237758	3.1151117341
O	5.0877783926	9.7694313030	3.6876996682
O	4.1461939205	7.3444249779	3.9643720776
O	8.7214027407	6.9192391653	3.9755142682
O	1.4183542712	23.5782001961	1.7452248722
O	1.6148990378	6.4155007314	1.1747306949
O	2.3341062981	9.0134931154	1.1006606734
O	6.3193668421	23.6556187403	1.2093230279
O	6.3011372611	26.3089435946	1.6432595511
O	5.7747554479	9.0256756241	1.1379550563
O	10.0726321859	25.0247521875	3.8781524870
O	10.1952257921	9.0782048090	3.7113725750
O	12.6986760084	17.3937261571	3.3603774808
O	13.4370458436	16.9697194692	0.8151673701
O	15.9336071220	17.0843978132	13.3519848920
O	18.3920941847	17.5530727850	0.8662304733
O	17.6924110399	17.6313865032	3.4574791280
O	15.2351402101	16.9553070646	2.7754617055
O	12.4985350995	12.5455707936	2.9470532837
O	13.6666637131	13.0602886504	0.6553251214
O	16.0820338892	13.1647079696	12.9724834700

O	18.2423334334	12.8338761866	1.1062874095
O	17.7104131182	13.1220362656	3.7021582297
O	15.1886018526	12.9411472358	2.8329903339
O	13.4555860142	14.9647413044	2.5881170566
O	18.1922644481	15.2817038982	2.2340317289
O	11.4166376136	18.8380868438	5.2073485204
O	11.5879610981	16.1891503228	5.5299678090
O	12.3015549676	13.5827369594	5.4024849559
O	15.6390817457	18.7469884528	4.7148697276
O	16.2631646069	16.1480290811	5.1074872376
O	15.6873637138	13.5686285552	5.4294340747
O	0.1347971527	17.0750487457	2.8075698538
O	20.1755205186	13.5597735982	2.7648072936
O	17.1158488852	14.5906457513	10.0139616303
O	16.3680815742	15.1117784725	7.5135040630
O	13.9878655517	15.4377274931	6.3256317574
O	11.7869272865	14.6969684610	7.7226226163
O	12.1100863913	14.9063399961	10.3981746824
O	14.5445508630	14.9028884047	9.4040478105
O	17.2427892460	19.4567526454	9.4527184618
O	15.9528142690	18.5374393878	7.3402072236
O	13.6990625648	19.4920986987	6.4004583785
O	11.4457341207	19.5028230695	7.8104883590
O	12.0717414856	19.1961659821	10.4068210617
O	14.5850609193	19.1741304448	9.5602386469
O	16.1877066596	17.0046775819	9.4850364295
O	11.6417787904	17.0663626624	8.8645978445
O	18.5058622611	13.3143298414	11.8659177361
O	18.0173572602	15.8804227211	12.2384546853
O	17.6296501077	18.5261883507	11.9249755160
O	13.8952344261	13.2460817464	11.3942723354
O	14.1317123508	15.8298788002	11.8907493485
O	14.0232404505	18.4817668785	12.1069216333
O	9.7620962107	15.1819993478	9.4506009109
O	9.6227935890	18.7127437671	9.5702516127
O	12.5100194203	25.7651342970	3.3075728337
O	13.5042647747	25.2094402248	0.8879324712
O	15.8850493776	25.2259365786	13.0683734634
O	17.9504446591	24.6130750579	1.2565947730
O	17.4180704054	25.3414708578	3.8241562515
O	14.9748338452	24.7124121553	3.0116176258
O	12.8059917345	9.3313237254	3.4951865377
O	13.8604944318	9.6452886427	1.1295769854
O	15.9976599236	8.8531976899	13.0867844213
O	18.2151875295	9.1795940776	1.1213059376
O	17.9217202966	9.3154017288	3.7944475819
O	15.4114118882	8.9282693648	3.2067729888
O	13.7530833178	7.2038533507	2.0637949405
O	18.2478130644	6.9433922383	2.5830056562
O	12.1414066670	23.7691577808	5.0353503768
O	11.6925364345	6.1033899086	5.6308260576
O	11.8145954908	8.7559079285	5.8746553502
O	16.0957806498	23.2769563118	4.9531460972
O	15.3226922251	25.7556560423	5.4455265269

O	16.1515630921	8.8890227671	5.8076949561
O	19.8591257140	25.2792274860	3.0142338203
O	0.2203845488	8.6893433029	2.7296380102
O	17.3626693171	6.8046958724	10.1012051326
O	15.8970386645	7.4225642860	7.9944665125
O	14.0665586162	7.2383544853	5.9975475932
O	12.3059365476	7.2872011082	7.9907946179
O	12.0807468600	6.9576308842	10.6201944971
O	14.7247854956	6.9203025316	10.2700488626
O	17.7816987714	23.1552245052	9.7947945443
O	16.4065945487	23.1302021468	7.5962324576
O	13.9973034924	22.7400081792	6.6233956244
O	11.6350735764	23.3868419152	7.6501387519
O	12.5158572788	22.9564809286	10.1621607971
O	15.1617758514	22.5468495977	9.9453139146
O	15.8432413106	25.0017903184	9.4312673065
O	11.7503918703	25.3783470208	9.4013946621
O	18.3353102577	8.6347036659	11.8539002923
O	18.3286723401	25.7820934082	12.2085455501
O	17.6384153322	23.2389262267	12.4597354312
O	13.5720888617	8.5844418164	12.0423532660
O	13.6158421353	5.9591587366	12.5034587239
O	14.0695729051	23.3886683154	12.3289890489
O	9.9456319139	7.6120089394	9.3012543996
O	9.9395241811	23.4618113171	9.7077820695
O	7.1104988968	14.9426160985	9.4244207514
O	6.2069080383	14.7809289407	11.9269575732
O	3.9294739529	15.1124435321	-0.0657723819
O	1.6544611917	14.6164958998	12.0367632121
O	2.0464600677	15.1338614230	9.4057331018
O	4.5780964502	15.5646362770	9.9929646288
O	7.2125871310	19.6708683428	10.2376362796
O	6.1802225015	19.0686980371	12.5924197633
O	3.7598538241	19.6174710429	0.1080693320
O	1.5664964249	19.5695627571	12.0213749250
O	2.1662739602	19.0264513663	9.4625301224
O	4.6284773998	19.0129525018	10.3943743171
O	6.5592808302	17.1747618748	10.7550516184
O	1.3683794104	17.0778063941	11.1466288710
O	8.5911407221	13.7759742545	7.5443998963
O	8.4652884776	16.4497833928	7.5769072151
O	7.7673365288	19.0454528416	7.6672213197
O	3.8370380022	13.6217107849	8.2646132933
O	3.6207879086	16.1958609556	7.5695253550
O	4.1667956111	18.8496805435	7.7123110231
O	19.6836518508	15.1418146002	10.3144725269
O	19.6962795857	18.9561927576	10.2496094725
O	2.7627260908	17.2470449193	3.0259810344
O	3.9260712992	17.5811305212	5.3502950406
O	6.0196228943	17.1166229177	6.9446713810
O	8.0528127395	17.8293311228	5.3235107895
O	7.7011960494	17.2228545526	2.7161771809
O	5.2798824860	16.4180845942	3.3328492312
O	2.5618850494	12.7348476284	3.5076870707

O	3.9729026669	12.7641237060	5.6955983829
O	6.1363587409	12.8414538089	7.2551596328
O	7.8301016314	12.8015681727	5.1768082078
O	7.7364506024	13.6053053588	2.6599579145
O	5.1685239443	13.3379407020	3.3864365038
O	3.3928713723	15.0602609519	4.4997490496
O	8.4365047584	15.3253171643	4.5579570842
O	1.4950798145	18.6450859445	1.1672830763
O	1.6311627872	15.9862830888	0.9164857131
O	2.1447426304	13.3620505232	0.9466533697
O	5.5701623761	18.5330466375	1.7633556736
O	6.2204001459	16.1350522387	0.8102817448
O	5.8521403583	13.4691947079	0.7973506530
O	10.0764019906	17.2382786206	3.6229793460
O	10.1138475308	13.3369556929	3.8586141376
O	8.8435595295	21.1890286367	8.8884827223
O	3.8006362758	21.1159289539	8.9685399448
O	5.8570174044	21.0119389053	1.0395809860
O	1.6765227834	21.2215492608	0.6525384564
O	1.0672625698	11.1467843996	2.0315143314
O	6.4946515105	11.3292788015	2.1118914218
O	4.1273657534	11.0897419596	7.7214084998
O	8.2455246518	11.1610411014	7.2063993262
O	11.3692297901	11.1617048027	4.8833951802
O	16.1196959694	11.1563233373	4.5733764074
O	14.5501485164	11.0195526392	12.4252281869
O	17.6095757223	10.9883434849	12.6829025458
O	18.2706436334	20.9793549349	11.3527186966
O	13.7419284782	20.9604451685	11.4412818201
O	15.9426426121	20.9447027829	6.1462574800
O	11.8065205823	21.3337193788	5.9448111749
O	7.0414888199	27.1586321009	10.2200890284
O	5.9765217866	27.4278546584	12.6157438140
O	3.8603151019	26.9475482918	0.7908902760
O	2.0873460013	27.4837639246	12.2270967620
O	2.0683996086	27.3628361043	9.5983743438
O	4.4582643943	26.6565326617	10.5331327358
O	3.7779128684	26.0741715299	8.0073786747
O	19.8012448766	27.5612645351	10.8987819573
O	4.3852196632	27.0979093375	4.1614505770
O	8.1699236545	26.8522937922	4.5419083365
O	1.3410024474	26.1438259617	0.9853680089
O	14.4687259480	27.2773963621	2.3966290830
O	18.2001041547	27.1246508820	1.9211855397
O	11.4671552356	26.2531800634	5.7535851028
O	17.3865263515	27.0808080034	10.0021295034
O	16.2473612973	26.9823826989	7.6310477309
O	13.7004251180	27.2155726408	6.8885544593
O	11.3322496659	27.6301049733	8.0267374926
O	12.1816407540	27.7409182818	10.5450532809
O	14.6996054432	27.3508222154	9.7406353542
O	13.6542685571	25.9078009043	11.7421072311
O	9.6542083855	27.0216071906	9.9486691063
O	2.6967995968	3.1745550673	10.0031745353

O	5.3558948078	2.8331749169	9.8220428137
O	3.9765964642	3.4407229427	7.6136603393
O	20.0764676602	3.4444482273	10.3853786201
O	17.5678197669	3.0382627159	9.8846706340
O	18.2819026800	3.3829965747	12.4330393900
O	7.5371424436	29.0612972502	3.2610791765
O	4.9854687770	29.6384452007	3.9706630503
O	5.6205464965	28.8874366987	1.4252681209
O	17.5323002868	28.9650094735	3.6898158927
O	15.0411200686	29.7752263270	3.2753721216
O	15.6181586579	28.4125790580	5.4763308842
O	6.0498198461	3.9232866874	1.0267402087
O	15.7407285918	3.7528218528	5.4544255322
O	16.1594614591	2.5056267618	7.7681898877
O	4.1846295045	28.6298332439	8.7203276729
O	3.5687568748	28.7881938020	6.0988196179
O	17.6636498330	28.3487755721	12.3035811622
O	6.4383788508	2.4263257519	12.2380697146
O	18.2779912214	29.6865488270	1.1453985846
H	7.2533763282	1.1134207709	8.2099954461
O	8.1238027492	1.1868707975	8.6299944478
Si	8.6468451398	2.7050436806	8.9267383664
O	7.9491573258	3.3439864998	10.2689478930
O	8.2388250139	3.6320452333	7.6335194245
O	10.2658465068	2.6589355700	9.1289351908
H	2.5590010297	0.9355962525	1.7738936757
O	2.0391837093	1.2218620203	1.0054628460
Si	2.4604865333	2.6744925717	0.3762494467
O	4.0174064394	2.6704330103	-0.1233832463
O	2.2398028308	3.8434720563	1.4909181076
H	13.8556402332	31.6468503294	11.7548011164
O	13.5723920887	31.0515908087	12.4735531256
Si	14.4019520132	29.7761813193	13.0219933741
O	15.9662534365	30.1112854970	13.3322675358
O	14.2969711482	28.4552008501	12.0859766022
H	12.4195273019	1.0528297404	12.0023974767
O	13.0432847839	1.1369299563	11.1919142263
Si	13.5967768581	2.6619873825	11.0380827779
O	12.4550431719	3.6548812814	10.3916050622
O	14.9400612050	2.6497609151	10.1151944283
O	13.9271599201	3.2718526652	12.5314656980
H	1.1693452489	31.4482339739	1.6525080362
O	1.4410091719	31.0386552925	2.4976126929
Si	1.4504528096	29.4005546413	2.5533918611
O	2.4203030161	28.8946663272	3.7562694692
O	1.9230118906	28.7901144036	1.1043091537
H	11.3648996672	31.5822635565	3.7633916017
O	11.3176808601	31.0219775247	4.5419775341
Si	11.4248449918	29.3859924244	4.3715066866
O	10.0505944546	28.6607267159	3.8510969768
O	12.5132107678	28.9061934341	3.2726851676
O	11.8867220116	28.9104571850	5.8631872930
H	11.6976228847	0.3528181527	6.2742608006
O	11.4522789805	1.2871829201	6.3172727327

Si	12.5907989562	2.4552779153	6.3660265592
O	14.0950263510	1.8144211546	6.1915411307
O	12.2872969359	3.5083001143	5.1479814656
O	12.5137938264	3.2959257529	7.7790879321
H	8.3623811055	31.6017267539	7.9673336740
O	7.6865184758	31.1651288740	7.4173337708
Si	7.6024916254	29.5649273591	7.1900234587
O	6.0311957984	29.1947774448	6.9235670525
O	8.2204657509	28.7342220034	8.4647211832
O	8.4276820686	29.1465295322	5.8449017638
H	8.1904231028	33.6733518411	2.9596731841
H	8.2279173580	30.9208257680	0.2138681093
O	8.3636504424	33.0497731504	2.2383326996
O	7.5985089710	31.6535017612	0.1339125071
Si	7.0528000479	32.3525567038	1.5168580635
O	5.8751713260	33.4309664550	1.1585113813
O	6.3174229083	31.2625162151	2.4826305505
H	21.5340893864	31.1267868221	11.9957097578
H	18.9755708550	30.9993893226	9.3287240972
O	20.7194290761	31.5813434815	12.3625265941
O	19.7287980506	31.0924217618	9.9371442368
Si	19.3979097614	31.7483914636	11.4377610300
O	18.9284147581	33.3080191187	11.2252348411
O	18.1242436373	30.9996185736	12.1665913137
H	1.1328553937	31.6083207657	8.5747390857
H	3.4560059138	33.9307528202	6.0365498780
O	1.4866659475	31.9084158883	7.7122089186
O	3.1830111279	32.9997048483	6.0315991939
Si	3.0783391914	32.2896401687	7.5050157190
O	3.6582790681	33.2510956453	8.7241429706
O	4.0691703996	30.9816081002	7.5532454020
H	19.3730927012	31.7514158015	4.0427411472
H	19.3798189313	32.0663384355	6.7978093087
O	18.5670537435	32.2895972911	4.0992469031
O	18.4346581475	31.8283305655	6.8013251472
Si	17.5860562231	32.1079981812	5.4070730617
O	16.6397515666	33.4366841925	5.4913071076
O	16.5087545982	30.8690359993	5.2497174164
O	20.0044777768	28.8117117889	2.9693476038
O	1.5532573760	2.9567994468	12.4397355052
O	13.6283615350	29.4559988354	1.0421640567
N	0.8438504156	19.6681642823	5.5719715356
H	-0.1682464961	19.7285130044	5.4392251323
H	1.2369459470	19.4749557618	4.6483164448
H	1.1570735122	20.6079872548	5.8243421515
N	10.4831902018	5.2736347662	0.2648746553
H	10.7223697575	4.6837557080	1.0649080061
H	10.9290949830	6.1761565820	0.4461166434
H	10.9773098265	4.8840555084	-0.5415651331
N	2.8483406907	30.8248018413	10.8343667456
H	2.7950384451	31.8069227178	10.5638333477
H	2.4030029993	30.2685651325	10.0968478700
H	3.8441579454	30.6002819118	10.7569424565
N	11.6512089145	0.7776225162	0.1160116585

H	10.6578310147	0.5674701988	0.2586830467
H	11.9199643132	1.4730683045	0.8124906104
H	12.1980673200	-0.0714590029	0.2922910118
N	19.7120038108	2.9332553885	5.7720305625
H	19.9960400826	2.2488285571	5.0664672360
H	18.9132088152	3.4060624462	5.3415240003
H	19.3389135157	2.3988005638	6.5601974775
N	9.4590250081	-0.0692103394	11.0375877967
H	10.2787306057	0.3603944215	11.4652777794
H	9.6066265058	-1.0791875591	11.0241105975
H	8.6645441459	0.1100789797	11.6559335557
N	4.8353066019	11.3539859585	11.6999072155
H	5.3509306562	12.0075684742	11.1055216021
H	5.4724084061	11.1196680563	12.4657018100
H	4.0763262812	11.8915331172	12.1272735804
N	9.1158575758	20.3389599002	1.4597135040
H	8.4296879083	20.3422994638	0.7030426058
H	8.7357905613	20.9714465742	2.1682710011
H	9.0907502641	19.4002361109	1.8644880907
N	15.7258839857	0.8031689984	1.7775898317
H	16.4222925420	0.5321753384	1.0798241136
H	14.9949719464	1.2974983787	1.2606523571
H	15.3182336415	-0.0764626843	2.1052456169
N	13.7112872164	30.7348309436	9.1551948363
H	13.1848257795	31.5096327018	9.5650793396
H	13.0333888797	29.9917148820	8.9647812432
H	14.0413332432	31.0690565031	8.2464440719
N	10.7441164478	27.6874498989	0.3767703407
H	10.0483712178	27.5533001760	1.1137168395
H	11.3254997178	28.4757250416	0.6686153137
H	11.3412001092	26.8583165141	0.4419373764
N	9.5142461650	24.9330793324	12.9178856930
H	10.3046195421	24.3703954017	12.5948617319
H	9.6526187123	25.8752924740	12.5403927257
H	8.6998948914	24.5443685750	12.4401919252
N	16.9960727631	10.7736263598	8.4421666851
H	16.8065757105	9.8135523144	8.7379361426
H	16.4507005363	10.9038422590	7.5874058646
H	16.5923349542	11.3894160164	9.1503311294
N	1.3325745814	11.5493096494	10.9871268641
H	2.1997345700	11.2639463587	10.5241374293
H	0.7359983781	11.9641452920	10.2671763599
H	0.8631621717	10.6829990498	11.2592167976

Table S2. Initial coordinates for the U0 MD simulation.

```
524
U0.INI.xyz
Si  6.387355831  3.510356871  10.94146632
Si  1.494848223  3.661407574  10.88840562
Si  1.564990102  4.941249059  2.109156211
Si  3.870814922  5.681986106  4.08823022
Si  4.414090962  4.944227152  7.11511519
Si  7.616105935  4.826946296  7.058112975
Si  8.588706218  5.36103057  4.120498642
Si  6.28436019  5.064931658  2.189438539
Si  11.59227463  4.803635732  4.560901564
Si  13.99816379  5.451789277  2.697260977
Si  14.44856389  4.70125667  13.07917859
Si  17.59707166  4.759390564  13.05132166
Si  18.50051036  5.332096593  2.58223638
Si  16.44550119  5.011958576  4.718536962
Si  16.00150458  3.201963366  9.277821447
Si  11.46252634  3.696833953  9.064901134
Si  8.350676489  7.305739438  8.838616495
Si  6.166204606  6.612013974  11.07517543
Si  5.560718133  7.535266584  0.481927775
Si  2.459413731  7.392349798  0.322722568
Si  1.422168891  6.831895402  10.8626789
Si  3.785498035  7.29107036  9.116706905
Si  8.522749569  22.73317893  9.291089652
Si  6.076512915  23.37741084  11.05694099
Si  5.425960786  22.48055119  0.579760556
Si  2.307744814  22.76710912  0.593383372
Si  1.40928451  23.7361642  11.09645374
Si  3.658572944  22.72137048  9.079320095
Si  1.346798481  25.16593487  2.267652643
Si  3.880485299  25.60479467  4.102081518
Si  4.321255938  24.93506153  7.1058737
Si  7.544875313  24.8668655  7.196436653
Si  8.538417487  25.37854463  4.338933069
Si  6.253651944  24.8246206  2.340685239
Si  1.495245011  9.544362904  2.370639254
Si  3.785387283  8.864180379  4.317211736
Si  4.48068686  9.614826852  7.16005986
Si  7.59005261  9.663470575  6.962359843
Si  8.625861238  8.574747038  4.156606748
Si  6.17544329  9.744922317  2.549119478
Si  11.46100142  17.38686603  4.411263085
Si  13.72765825  16.57418648  2.354958054
Si  14.39479132  17.08785866  12.83704882
Si  17.49857825  17.24763376  12.91604713
Si  18.61463716  16.84484966  2.385610427
Si  16.18025662  17.47300138  4.000790646
Si  11.49624817  12.64022213  4.283428953
Si  13.71278343  13.38313142  2.279372053
Si  14.47467416  12.58875815  12.75985553
```

Si	17.57205068	12.596522	12.95772159
Si	18.52889961	13.68319856	2.445082866
Si	16.13359056	12.67167939	4.133348691
Si	18.28997551	14.77092457	11.03432566
Si	16.04893545	15.44054584	9.016780601
Si	15.51598124	15.11532561	6.004996404
Si	12.37723102	14.96485222	6.206067414
Si	11.34715478	15.49061905	9.082002176
Si	13.70296959	14.71954696	10.7845074
Si	18.2725068	19.50190871	10.81512095
Si	15.98578146	18.57320659	8.985213686
Si	15.34520851	19.5175648	6.164226933
Si	12.1599672	19.76122407	6.321334746
Si	11.22676273	18.63251703	9.136960245
Si	13.62970342	19.45786539	10.85586634
Si	11.52036542	25.24383647	4.55640804
Si	13.85349146	25.79130177	2.451810655
Si	14.3035408	24.86258314	12.93680452
Si	17.45729055	24.77329603	13.07161848
Si	18.41320746	25.60024478	2.467744857
Si	16.0579038	24.86039017	4.330526225
Si	11.50285953	9.532145563	4.457451679
Si	13.93239468	8.654160658	2.595365744
Si	14.4619303	9.456009947	13.01578938
Si	17.56248023	9.434410858	13.05127224
Si	18.58324464	8.532642095	2.58612189
Si	16.35908344	9.605841965	4.444139503
Si	18.44493522	7.135123205	11.25210214
Si	15.96116237	6.402389901	9.339971187
Si	15.67531397	7.446637574	6.387789639
Si	12.48066675	7.313512928	6.366105053
Si	11.28821532	6.760274895	9.174981568
Si	13.51989849	7.093745881	11.28527945
Si	18.4322294	22.62555724	11.12778439
Si	16.23256258	23.51011159	9.201147787
Si	15.57220894	22.61318073	6.296341579
Si	12.37766794	22.80703097	6.345407058
Si	11.47193119	23.73797917	9.282690589
Si	13.84966706	22.5202988	11.03536453
Si	8.451745734	15.09017558	8.535268279
Si	6.088175231	15.58289234	10.56783113
Si	5.545857779	14.82472433	0.053440364
Si	2.34971878	14.77658049	0.172642751
Si	1.192817407	15.5298087	10.79617372
Si	3.526473662	15.05806405	8.834884891
Si	8.371666668	19.62935102	9.070736393
Si	6.075802177	18.76566202	10.98775829
Si	5.325681979	19.52988811	0.527117017
Si	2.145072728	19.75200257	0.212280572
Si	1.212160644	18.67390437	10.79142224
Si	3.659881106	19.55973285	9.113338314
Si	1.549353907	17.26837161	2.037641537
Si	3.794181302	16.5648449	4.114151635
Si	4.406501865	17.42466003	6.977142618

Si	7.556365779	17.57534484	6.90611382
Si	8.519718636	16.85254008	4.09516234
Si	6.154465292	17.04452245	2.173611969
Si	1.444882592	12.6503806	2.292789825
Si	3.735794902	13.48058018	4.22149107
Si	4.432917027	12.58130077	7.173725449
Si	7.586054896	12.6360207	6.879963678
Si	8.488358033	13.71333963	4.129512031
Si	6.233110554	12.88925672	2.274373002
Si	8.270543071	27.31559507	9.086985446
Si	5.954780608	26.56072291	11.1506805
Si	5.481965236	27.39349008	0.67348179
Si	2.305553557	27.47961633	0.437391074
Si	1.253618957	26.95063539	10.92628473
Si	3.646836275	27.28725506	9.176640152
Si	3.92074746	28.68567574	4.401052751
Si	8.560266922	28.51903226	4.410324629
Si	13.93469858	28.93032491	2.516361879
Si	18.49448335	28.63976423	2.518953599
Si	18.27504981	27.27198183	11.36485808
Si	16.0010811	26.68443385	9.262238789
Si	15.25762281	27.19513341	6.363121911
Si	12.1249128	27.52646005	6.614631115
Si	11.23001625	26.901156	9.501541988
Si	13.66776702	27.27878493	11.13450756
Si	3.908024568	2.673809031	9.03855241
Si	18.64213443	2.700198976	10.99826287
Si	6.221004399	29.71084156	2.64818053
Si	16.22111609	29.52195741	4.429749088
Si	5.592927197	2.585199675	0.315131108
Si	15.65694863	2.433485036	6.295098075
Si	4.581921164	29.52794086	7.275642086
Si	17.54539228	29.81453778	13.20175728
O	6.139428575	5.034187719	11.45062304
O	1.743844467	5.251599417	11.03575455
O	8.339052672	6.141237912	7.703374963
O	2.471108464	5.226766818	3.418070566
O	3.869106122	5.165968507	5.61281637
O	6.020835874	5.089409731	7.222321402
O	8.001576353	4.755253213	5.489679082
O	7.739509852	4.761338704	2.866129691
O	5.141912774	5.019677588	3.33909235
O	6.34786607	6.511886006	1.480777368
O	10.12051177	4.895183121	3.88738166
O	12.61375632	4.890223892	3.303745129
O	14.28643403	4.692449287	1.300339228
O	16.02131931	4.818328791	12.64823026
O	17.75206028	4.693245402	1.279424214
O	17.81573921	4.694043952	3.908643244
O	15.21412092	5.082517787	3.680509784
O	16.55904002	6.429826572	5.486513873
O	0.024607476	4.881403085	2.609931735
O	15.82183395	4.810914118	9.031992264
O	10.94348372	5.216674789	8.835364289

O	18.35469052	6.091009908	12.50702994
O	7.420872356	6.909795049	10.09979279
O	6.326375328	7.501728583	12.4280473
O	4.031820451	6.983944901	0.341771636
O	1.799079525	7.577372996	12.24486737
O	2.265887676	7.499795346	9.646065374
O	4.749333974	6.960742691	10.36836915
O	7.544105655	22.86689136	10.57970748
O	5.670812858	22.52272894	12.36671763
O	3.882564315	22.79072928	0.945310267
O	2.140174637	23.39403157	12.49678379
O	2.333745743	23.18888454	9.888564569
O	5.007574348	23.05086119	9.897188992
O	6.012121924	24.95629155	11.37751341
O	1.202663055	25.3337914	10.98535617
O	7.810189537	8.710718969	8.23840589
O	8.146625055	26.20687746	7.896005104
O	7.87372814	23.52968287	8.042870221
O	4.337778707	8.6495851	8.437225733
O	3.740668636	6.094354821	8.035018786
O	3.748692643	23.52413551	7.674898565
O	19.87518044	7.034801817	10.5043873
O	19.99961785	22.99255522	11.03779592
O	2.428554023	25.48192957	3.412352379
O	3.728853166	25.07022805	5.614053705
O	5.941335288	24.99783511	7.032963945
O	8.270267535	24.65876317	5.764320031
O	7.605754188	24.72814117	3.205757183
O	4.942748185	24.6802774	3.290092226
O	2.460493225	9.513202393	3.66726599
O	3.607889329	9.046777856	5.918741945
O	6.007155034	9.717333998	6.626482162
O	8.353164104	9.108443785	5.658736937
O	7.602290592	9.18784326	3.071642701
O	5.082754295	9.654725093	3.749586853
O	3.991679394	7.297411178	3.953328829
O	8.47744708	6.970802442	4.147501256
O	1.52235188	23.66594483	1.677566614
O	1.677754231	6.119387564	1.005188928
O	2.200695523	8.767478769	1.141570918
O	6.348399687	23.56792142	1.324412092
O	6.205800732	26.21697556	1.52212832
O	5.675401181	8.98353262	1.207067031
O	10.06676522	25.12959052	3.867142174
O	10.12893342	9.033660431	3.750109782
O	12.61230912	17.29173932	3.279772863
O	13.47619446	16.89661125	0.78357744
O	15.93886025	17.16178093	13.3187525
O	18.41706117	17.36383552	0.861144379
O	17.68016057	17.68332536	3.415530601
O	15.18582617	17.15896601	2.759778216
O	12.41044577	12.67224995	2.938836379
O	13.67210624	13.1329621	0.68304268
O	16.020185	13.06422402	12.91149818

O	18.16385455	13.06282394	1.004132144
O	17.62880082	13.00659674	3.609365079
O	15.08556544	12.74303348	2.884592964
O	13.67937475	14.9715049	2.621380311
O	18.22265053	15.27456343	2.471632491
O	11.54245817	18.82311925	5.15881143
O	11.54925042	16.16266587	5.484461966
O	12.20883357	13.55384209	5.424066428
O	15.73712189	18.83564253	4.752037145
O	16.16576704	16.22095104	5.026701875
O	15.69134426	13.64859779	5.348789663
O	0.135758087	17.09009097	2.816178304
O	20.09543848	13.39796226	2.744714792
O	17.2115774	14.6665379	9.832654254
O	16.28410505	15.20775305	7.432766785
O	13.93768529	15.41819973	6.226226887
O	11.81304723	14.74897226	7.710010804
O	12.16132571	14.95002173	10.36946895
O	14.61009976	14.81142099	9.432923172
O	17.2702494	19.37622473	9.550266286
O	16.01709486	18.65079304	7.366110322
O	13.75162914	19.49920233	6.419754535
O	11.48023695	19.44506407	7.757653241
O	12.10400323	19.23424021	10.35747653
O	14.6183398	19.23231684	9.571221714
O	16.08629226	17.01472711	9.43316366
O	11.61533554	17.08071098	8.895264285
O	18.41406026	13.33398401	11.77681302
O	17.88441123	15.89652552	12.11821387
O	17.75756756	18.57334589	12.03460187
O	13.81819623	13.24781972	11.43621203
O	14.21346992	15.81950739	11.85133928
O	14.00312379	18.44001515	12.05141282
O	9.801745666	15.15139975	9.426447178
O	9.66857005	18.77740604	9.55989228
O	12.54347718	25.73413641	3.401273515
O	13.54206605	25.20103097	0.959959548
O	15.88739657	25.19282695	13.10918347
O	18.01545729	24.6626897	1.203958777
O	17.49211377	25.32925655	3.767018301
O	15.01763472	24.85694824	3.081597554
O	12.6848405	9.253143991	3.405609689
O	13.87132378	9.31520417	1.130080323
O	16.01060647	8.967788896	13.03300233
O	18.15479565	9.11423225	1.138839795
O	17.82474159	9.301947502	3.806608089
O	15.30319664	9.049686391	3.348592075
O	13.87661307	7.043956303	2.470375364
O	18.28255293	6.93838125	2.607912525
O	11.94857634	23.80286687	5.149363774
O	11.81061135	6.02972447	5.617923269
O	11.81706863	8.697530861	5.818422723
O	16.14450229	23.37388021	4.977047387
O	15.55078758	25.87480267	5.48237225

O	16.14485912	8.924224029	5.906184338
O	19.91253094	25.26503013	2.955920435
O	0.138829685	8.779199759	2.807332969
O	17.30099117	6.766928857	10.17224604
O	16.01271251	7.244210996	7.954205318
O	14.08458883	7.242485563	6.085538286
O	12.22015852	7.311284207	7.960493116
O	12.07480815	6.967906393	10.57453885
O	14.67793754	6.905861904	10.17466252
O	17.70233013	23.12905559	9.767258958
O	16.29232854	23.19232946	7.621929301
O	13.986049	22.85312397	6.487527665
O	11.73567698	23.25940442	7.758759038
O	12.48077947	22.99614544	10.30583794
O	15.08526031	22.66090199	9.977165187
O	15.89007091	25.07676095	9.434052818
O	11.67888751	25.34577581	9.367958507
O	18.33998528	8.641644277	11.86102156
O	18.27862857	25.87230331	12.19109219
O	17.68408873	23.32021976	12.39316842
O	13.6153475	8.56365029	11.96791542
O	13.66857885	5.971263552	12.44999251
O	14.08512935	23.32925457	12.43710188
O	9.885188739	7.565154563	9.301241828
O	9.948477264	23.38786239	9.708882811
O	7.162289364	15.02098729	9.504536862
O	6.25955679	14.80962484	11.98092167
O	3.934946135	15.01345037	-0.067588455
O	1.606514251	14.6691198	12.11492589
O	2.064650393	15.14362739	9.502073735
O	4.596598685	15.28341006	10.02872037
O	7.269007962	19.5679139	10.2514241
O	6.141139796	19.0971245	12.57299568
O	3.752117133	19.5699765	0.136846449
O	1.538780335	19.5605007	12.10612281
O	2.195181891	19.03297555	9.561577332
O	4.658051474	19.24401329	10.36310832
O	6.281703044	17.17604933	10.76609664
O	1.336836064	17.10153737	11.17873049
O	8.485085538	13.74785696	7.62921674
O	8.465544795	16.40700135	7.577913383
O	7.801331667	18.98145783	7.686847071
O	3.669988583	13.59909406	8.161620728
O	3.669128832	16.18503385	7.694989918
O	4.138959865	18.82346771	7.751071805
O	19.70342643	15.15488461	10.36605767
O	19.73271685	19.02560469	10.29653822
O	2.728816436	17.29208036	3.140036518
O	3.943313436	17.49865898	5.426148368
O	6.00900657	17.10809966	7.026078855
O	7.999341713	17.75511693	5.351587981
O	7.661669753	17.15403465	2.75264314
O	5.219812224	16.42888557	3.346091071
O	2.476452585	12.7352733	3.535590298

O	4.000358121	12.74782848	5.628168598
O	6.033094299	12.81495388	7.30359954
O	7.742171794	12.82187005	5.26971737
O	7.658919684	13.53010362	2.743318919
O	5.058581871	13.34980089	3.293988634
O	3.345198139	15.04426785	4.479165883
O	8.470221431	15.28597192	4.545735757
O	1.526706025	18.68740122	1.258102446
O	1.739584199	16.03278375	1.000727895
O	2.074356053	13.39997314	0.987793795
O	5.602442526	18.49981086	1.739338095
O	6.150049933	16.0842773	0.873787804
O	5.878545807	13.42307814	0.785510936
O	10.02176239	17.29007491	3.677946377
O	10.01563997	13.20745773	3.939947132
O	8.741759681	21.1885386	8.833498055
O	3.596837198	21.13546274	8.77120734
O	5.811535147	20.98030118	1.070907125
O	1.735560913	21.25013621	0.679986541
O	1.193055592	11.08509826	1.936713613
O	6.346451908	11.2955014	2.148568332
O	3.999695227	11.0883769	7.636325026
O	8.128617987	11.16303421	7.297521158
O	11.4106514	11.11271883	4.8232565
O	16.14916872	11.17872637	4.739585966
O	14.34045145	10.99855546	12.58359884
O	17.75787285	11.00884011	12.78208304
O	18.28751994	21.03111466	11.32243368
O	13.75641849	20.95905172	11.44090264
O	15.8976339	21.03678006	6.152066864
O	11.8750751	21.31510743	5.952865305
O	7.033834429	27.14422417	10.10356128
O	6.011051354	27.41017453	12.52836176
O	3.870436243	27.13320092	0.672708143
O	1.939754185	27.60339285	12.24914952
O	2.07994885	27.41798206	9.615819749
O	4.477215081	26.86866774	10.50433197
O	3.796766179	26.15123414	8.03170124
O	19.7652466	27.54721025	10.77809192
O	4.395465841	27.15044704	4.078367023
O	8.208227025	26.94694871	4.489175796
O	1.417033497	26.26753747	1.066412652
O	14.34843822	27.34738246	2.398033334
O	18.2804201	27.13469947	1.94405767
O	11.41328086	26.3399582	5.759492201
O	17.28043878	27.21816183	10.09039788
O	16.1601989	27.14071823	7.712741517
O	13.69118197	27.15851703	6.766427286
O	11.47782968	27.66948325	8.089016585
O	12.14808557	27.61856663	10.64770867
O	14.62678608	27.34619393	9.824059333
O	13.67254356	25.79007055	11.77040133
O	9.67129806	27.07495512	9.898413141
O	2.546413999	3.075446829	9.828510776

O 5.214215998 3.081177361 9.90627612
O 3.934954359 3.487746054 7.627910261
O -0.022084186 3.354178307 10.37529235
O 17.46980715 2.951788616 9.917689342
O 18.27614002 3.416634401 12.43070328
O 7.597633909 29.15773957 3.293304735
O 5.020196323 29.68507761 3.749716685
O 5.806489865 28.82538366 1.343567664
O 17.55290465 28.8254212 3.82088385
O 15.15317223 29.75129355 3.21496174
O 15.55758668 28.54990131 5.548629542
O 6.00325487 3.952564962 1.068841039
O 16.21665769 3.879469772 5.825802061
O 15.94350831 2.350323459 7.894468244
O 4.134953341 28.73056887 8.630284587
O 3.790572998 28.88959044 6.000765033
O 17.83029146 28.48293593 12.32642603
O 6.337217035 2.546114029 12.25044706
O 18.14979543 29.6846781 1.32730142
Hm 8.606742647 0.256237304 10.03164975
Om 8.136343129 1.040348846 9.032748595
Sim 8.545165795 2.574784926 9.032921477
Hm 1.935463568 0.427648033 1.365712615
Om 1.867916683 0.953559328 0.537016985
Sim 2.39646733 2.487754704 0.310795075
Hm 13.61006321 30.88284494 11.13745812
Om 13.67361202 30.89839653 12.18568786
Sim 14.37888507 29.72658019 12.99923618
Hm 12.24097203 0.982129965 12.03054073
Om 12.74549781 1.25596824 11.17726612
Sim 13.44381612 2.692740583 11.08779649
Hm 0.61181439 31.49271161 3.00842137
Om 1.33427091 31.09795663 2.477738813
Sim 1.443959153 29.46000739 2.597748598
Hm 10.46911958 31.47886906 4.035589451
Om 11.21522853 31.11304367 4.54636342
Sim 11.42930007 29.48945572 4.428654469
Hm 11.70129409 0.354635139 5.832797167
Om 11.81364365 0.999544343 6.562826519
Sim 12.50408795 2.447628925 6.398622711
Hm 7.981063131 32.02300094 7.977071185
Om 8.018752312 31.26195097 7.306857837
Sim 7.719565743 29.71068994 7.215255377
Hd 8.910886968 1.425862636 1.261187143
Hd 6.248589287 -0.737692146 -0.919995532
Od 8.625955452 0.472292935 1.524996927
Od 7.137250905 -0.59982079 -0.401397819
Sid 7.116638314 0.057404911 1.09577146
Hd 21.16351238 32.08826986 12.62260565
Hd 19.47579251 30.15447911 9.846079298
Od 20.82459678 31.57529558 11.84680675
Od 19.07961376 31.03661778 9.959358321
Sid 19.26509317 31.74375747 11.42293926
Hd 1.635586965 -0.476221151 9.137775729

Hd	2.188062502	1.408551168	5.982416064
Od	1.733113672	-0.402980845	8.1727919
Od	3.004133272	0.799643377	6.161656655
Sid	3.179683859	0.121443478	7.597150891
Hd	17.70352218	0.69279271	2.862980418
Hd	18.61563762	-0.330476644	7.066006327
Od	18.22097058	0.332295739	3.682766227
Od	18.73915192	0.004546572	6.161570683
Sid	17.49428458	0.096507468	5.101031825
Or	7.868044339	3.409175841	10.301445
Or	8.081819065	3.421870349	7.697626465
Or	10.17377408	2.742599754	9.236142707
Or	3.875476418	1.085067347	8.764593137
Or	18.81725864	1.130246684	11.302621
Or	12.42130691	3.755150948	10.37831054
Or	14.83528724	2.616588727	10.236094
Or	13.81578259	3.295626094	12.57056393
Or	2.466552672	28.9596472	3.745445759
Or	1.935242242	28.90299245	1.141992276
Or	20.02469167	28.83143902	3.00958815
Or	6.471579313	31.2265722	2.205046305
Or	10.09117986	28.74707727	3.87565448
Or	12.59974071	29.08720271	3.397567434
Or	11.88494714	28.96337541	5.89859503
Or	16.56168624	30.91239584	5.156539606
Or	6.077857521	1.330313687	1.184638098
Or	3.993706057	2.543716206	0.07141752
Or	1.990117936	3.481027458	1.532405651
Or	16.42584391	1.273892303	5.492001918
Or	14.07341862	2.341816402	5.960889863
Or	11.74309887	3.36311202	5.279083283
Or	12.41373522	3.212879824	7.832281666
Or	4.275217203	31.08381144	7.491673215
Or	6.157372691	29.28749103	7.02823956
Or	8.283002309	28.83156211	8.500346007
Or	8.479908436	29.16120337	5.878525361
Or	18.22619456	31.11034921	12.52090446
Or	15.94851517	30.02917428	13.31535572
Or	14.22727093	28.28524376	12.250766
Or	1.660512389	2.947685607	12.33017666
Or	13.62198112	29.56953148	1.063296888
N3	0.937738281	18.09219107	6.554173312
H3	0.421045826	17.65651939	5.781814521
H3	1.158635462	19.04012018	6.229525728
H3	0.257978997	18.21575501	7.312568829
N3	9.36550974	2.951211258	0.749614978
H3	8.988164615	3.63567552	1.417081647
H3	8.962041462	3.177049973	-0.167247985
H3	10.37012873	3.142655151	0.674247835
N3	4.937107682	31.23335635	11.48518645
H3	4.041188814	31.35235112	11.97267941
H3	4.908627141	30.29307593	11.07323259
H3	4.918676372	31.88992775	10.69324452
N3	11.59661579	0.405365554	0.068331813

H3	12.08953293	-0.496597109	0.036013635
H3	10.63383788	0.237790048	0.391539491
H3	12.05153744	0.944977209	0.813139202
N3	20.85644581	2.284165264	5.754592144
H3	20.70353377	2.689284508	4.825498755
H3	20.77583733	3.055825358	6.424437699
H3	20.07833594	1.634880284	5.935858019
N3	9.010862134	-0.483746818	10.85841159
H3	9.944460695	-0.18104978	11.16028861
H3	9.088194662	-1.428605642	10.46288329
H3	8.370311605	-0.516455546	11.6908485
N3	4.050492164	9.810146894	12.16201556
H3	4.593643717	9.830923506	11.29154436
H3	4.499324723	10.50025936	12.77436703
H3	3.122814388	10.20271439	11.91830112
N3	9.212956594	19.77173341	0.80250717
H3	8.702897872	19.48726494	-0.040042796
H3	8.577640869	20.39336733	1.31412308
H3	9.302045317	18.92770222	1.378343954
N3	16.95229217	1.272724997	1.532353449
H3	17.61978635	1.56605942	0.809626508
H3	16.38001375	2.097040724	1.745380638
H3	16.3345154	0.583984428	1.088296967
N3	13.4313879	30.94710022	9.572159435
H3	12.98586413	31.86543435	9.454253738
H3	12.83404637	30.24926955	9.115617183
H3	14.30937311	30.97541037	9.043437862
N3	10.74621613	27.37926008	0.743194106
H3	10.54548353	27.7197217	1.689830606
H3	11.34624697	28.08697025	0.308161086
H3	11.32651863	26.5419259	0.856317784
N3	8.670899568	25.61065688	12.50036341
H3	8.449297821	24.92185645	13.22823529
H3	9.270983473	26.31584684	12.95990281
H3	9.273641121	25.11196528	11.83700853
N3	16.14803399	11.28611838	9.834205734
H3	16.31438073	10.44470816	9.270780114
H3	16.76238372	12.00632538	9.438540258
H3	15.19307108	11.58486016	9.606908781
N3	1.276678081	11.06001799	11.1796235
H3	1.426592822	11.19964267	10.17403279
H3	0.997554264	11.97552275	11.54899828
H3	0.44584148	10.46298923	11.25879287

Table S3. Initial coordinates for the U7 MD simulation.

```
524
U7.INI.xyz
Si  6.442789656  3.488749987  10.93068669
Si  1.497923256  3.722966938  10.88398414
Si  1.549610905  5.074779421  2.211219766
Si  3.90337794  5.680930517  4.109026778
Si  4.401426924  4.943806676  7.151219771
Si  7.598749875  4.794274799  7.072334317
Si  8.60640375  5.348560773  4.119545431
Si  6.288457325  4.925584008  2.157228986
Si  11.61276685  4.827162912  4.573704421
Si  13.94149539  5.611602518  2.551960366
Si  14.43205173  4.613824573  13.05876093
Si  17.59338784  4.816882458  13.02974661
Si  18.48488634  5.354402083  2.610481945
Si  16.29568133  5.036369263  4.596954272
Si  16.16998974  3.351284207  9.193872581
Si  11.4708927  3.59803425  9.185773558
Si  8.400526051  7.269645652  8.832492877
Si  6.212753888  6.589823522  11.05347492
Si  5.552369785  7.45157511  0.465703613
Si  2.435574861  7.435722965  0.322642087
Si  1.431567528  6.882198745  10.79759836
Si  3.81818238  7.286289045  9.112365704
Si  8.463365718  22.76753558  9.354068038
Si  6.09737865  23.4529252  11.09854402
Si  5.289513722  22.60356313  0.628448327
Si  2.19880512  22.6924869  0.582842399
Si  1.412079664  23.67174609  11.0686074
Si  3.712969425  22.75220139  9.088679587
Si  1.245254177  24.98371454  2.298072594
Si  3.68370042  25.55179825  4.142152292
Si  4.288683473  24.93784554  7.143540161
Si  7.476016139  24.89333025  7.253996809
Si  8.529106981  25.30355648  4.447541514
Si  6.248188546  24.87989046  2.48013829
Si  1.544917378  9.530513219  2.384768095
Si  3.893892851  8.865538242  4.353913904
Si  4.556464432  9.61952587  7.194236
Si  7.652455672  9.648163969  6.929936451
Si  8.714946837  8.573085344  4.117054608
Si  6.241309678  9.710317999  2.54534489
Si  11.48309677  17.38762081  4.426967691
Si  13.75704588  16.60858  2.369016811
Si  14.34016681  17.18702873  12.83995284
Si  17.48320525  17.26241527  12.93292501
Si  18.59585815  16.84735045  2.43144172
Si  16.18891099  17.4736649  4.067322613
Si  11.5260516  12.61323156  4.324146751
Si  13.74809544  13.39199274  2.305453373
Si  14.4915693  12.5693015  12.77811806
```

Si	17.60043787	12.6354117	12.99735483
Si	18.56958199	13.68959768	2.482760259
Si	16.18850944	12.65203186	4.164742224
Si	18.28000627	14.78486414	11.06288896
Si	16.00368744	15.450342	9.077964351
Si	15.53889394	15.08202864	6.052460419
Si	12.36587349	14.9666271	6.246534289
Si	11.33670342	15.545648	9.134371055
Si	13.65895446	14.75235837	10.84328981
Si	18.27948371	19.51178739	10.8193761
Si	15.95156228	18.58511617	9.046331299
Si	15.33903122	19.52734177	6.224058833
Si	12.1414014	19.77548693	6.334237765
Si	11.2170971	18.67935329	9.178690663
Si	13.5958801	19.57766703	10.89152916
Si	11.58656022	25.18137666	4.431342928
Si	13.87111761	25.55958664	2.341077917
Si	14.28458633	25.0801622	12.76100087
Si	17.44750887	24.75922086	13.07108314
Si	18.29451323	25.5990552	2.500200134
Si	15.93916129	24.72760994	4.28806542
Si	11.58112325	9.510881879	4.501395994
Si	13.96865114	8.712513783	2.512353216
Si	14.56451497	9.492727976	13.03319803
Si	17.65206681	9.475067839	13.13465179
Si	18.65600538	8.543117992	2.651348657
Si	16.36470729	9.546790663	4.401480894
Si	18.48445488	7.258564891	11.24393235
Si	16.02182203	6.549595845	9.384220561
Si	15.61103381	7.389346632	6.406250268
Si	12.43835976	7.256964996	6.418916522
Si	11.3265377	6.731454455	9.301819099
Si	13.54495052	7.046183131	11.35361252
Si	18.42724029	22.626641	11.11687199
Si	16.22857575	23.53998825	9.186329849
Si	15.54392107	22.59619483	6.348881967
Si	12.35024248	22.8165732	6.2555951
Si	11.39986868	23.80698875	9.08696027
Si	13.78189943	22.60668498	10.80838455
Si	8.447161425	15.11706087	8.550421267
Si	6.089990224	15.60629735	10.60805288
Si	5.533260898	14.84347663	0.077668564
Si	2.341070718	14.74085581	0.159845927
Si	1.182265143	15.52990192	10.76982583
Si	3.541096162	15.07236238	8.847037421
Si	8.355294068	19.67146577	9.038177403
Si	6.084513022	18.77794702	11.02275398
Si	5.360710336	19.60947905	0.529578228
Si	2.184174817	19.66794761	0.17064677
Si	1.218517429	18.65529253	10.72769426
Si	3.693598616	19.55472719	9.148882178
Si	1.548094647	17.20915214	2.054017369
Si	3.817589177	16.5620677	4.142194608
Si	4.460327447	17.4418257	6.998365848

Si	7.611825006	17.60238138	6.877273957
Si	8.57271699	16.85125344	4.0538857
Si	6.170893055	17.09767677	2.162089942
Si	1.476507036	12.64856979	2.282445527
Si	3.786611893	13.45008122	4.272167653
Si	4.465065031	12.55914576	7.209893604
Si	7.626235324	12.63712742	6.893462328
Si	8.544955297	13.69505427	4.108837408
Si	6.253541673	12.84989161	2.300789031
Si	8.303927687	27.393543	9.046574588
Si	6.082116622	26.63238708	11.20087765
Si	5.583916342	27.36706411	0.748242768
Si	2.465529229	27.32889301	0.5584776
Si	1.283937487	26.89349984	11.05129213
Si	3.696089728	27.23907424	9.293879293
Si	3.707770624	28.69818898	4.383672439
Si	8.569440183	28.416876	4.359382325
Si	14.06285631	28.59394758	2.424037215
Si	18.51500758	28.66436563	2.341531918
Si	18.27844332	27.2213916	11.32657306
Si	16.08291272	26.63817673	9.221376162
Si	15.32475781	27.1050507	6.290058072
Si	12.22361263	27.59353081	6.419704056
Si	11.2870543	26.97322388	9.302536323
Si	13.74605888	27.7019471	10.98594235
Si	3.916534294	2.66136112	9.102362189
Si	18.66922003	2.695731836	11.02097653
Si	6.183821866	29.65427771	2.701872291
Si	16.30073523	29.47604927	4.384327547
Si	5.539726505	2.472117968	0.242961424
Si	15.54761798	2.418733001	6.234642473
Si	4.554711146	29.43538441	7.249647846
Si	17.53205652	29.81922946	12.94618362
O	6.271569322	5.014890197	11.45222903
O	1.801081163	5.304474859	10.76317703
O	8.395098182	6.031675539	7.773112637
O	2.418013578	5.374838704	3.548223227
O	3.947986514	5.079670557	5.610148045
O	6.00793488	5.085971433	7.301395823
O	7.930318511	4.821324683	5.489893959
O	7.732188408	4.747532674	2.889205579
O	5.060702028	4.956629894	3.228321927
O	6.333060665	6.307229514	1.329524211
O	10.10850055	4.76219142	3.954753646
O	12.62819773	5.099886096	3.343723834
O	14.15305793	4.62981084	1.276667148
O	16.00417893	4.901105478	12.71421125
O	17.81883413	4.76480681	1.249419968
O	17.7217681	4.696401925	3.88844782
O	15.26790571	5.566000407	3.467283752
O	16.4884701	6.224658056	5.690681697
O	0.009125917	4.886555131	2.680582024
O	16.22201385	4.973564559	9.095746821
O	11.01535749	5.150057405	9.292782988

O	18.35029104	6.103649391	12.38711054
O	7.464065974	6.965208908	10.10916804
O	6.273133112	7.505734754	12.39081987
O	3.996995258	6.975476307	0.3449061
O	1.746174914	7.506857736	12.25057416
O	2.339590261	7.637600153	9.679518164
O	4.79667445	6.827903123	10.30731544
O	7.538430528	22.79051196	10.70290867
O	5.480580045	22.72196301	12.40158397
O	3.744112979	22.85513445	1.066478206
O	2.145048324	23.25457075	12.4467833
O	2.339623852	23.19545923	9.82954584
O	5.017768414	23.26002654	9.909198442
O	6.333259999	25.03979638	11.3701392
O	1.204766686	25.27757469	11.04279781
O	7.87866795	8.648472461	8.166137288
O	8.069934263	26.26588156	7.899981813
O	7.672472236	23.61878349	8.226062608
O	4.469401064	8.596271208	8.431089232
O	3.671381718	6.094533761	8.020916267
O	3.80820309	23.45806158	7.624628741
O	19.88771093	7.1061641	10.44099942
O	20.00702472	22.92914427	10.96908991
O	2.337991859	24.94578332	3.484399252
O	3.598692393	25.26035279	5.727226604
O	5.894720612	25.06799241	6.960978884
O	8.284385095	24.58976178	5.885893555
O	7.593122206	24.60883303	3.32959345
O	4.964585569	24.80669168	3.472966884
O	2.593039578	9.453709813	3.612690839
O	3.660177778	9.113797421	5.941989652
O	6.066528999	9.720394094	6.622531714
O	8.408603369	9.111004787	5.613365305
O	7.699469798	9.188438933	3.018743188
O	5.208049331	9.647486413	3.803362098
O	4.144491449	7.28500667	4.079127534
O	8.604450193	6.963852246	4.085247498
O	1.210241159	23.53985379	1.544256334
O	1.606447894	6.283351152	1.139814879
O	2.200983697	8.851074718	1.072765245
O	6.202483065	23.71718275	1.351228604
O	6.348059684	26.33154565	1.741579759
O	5.728086422	8.851270908	1.26998315
O	10.04718189	25.07591804	3.946635959
O	10.22129823	9.040108392	3.742661746
O	12.67270137	17.29956548	3.341953075
O	13.47149358	17.02655816	0.825205566
O	15.90255254	17.22671468	13.25616739
O	18.34440816	17.36431343	0.9158669
O	17.68806616	17.68435208	3.485368667
O	15.2184782	17.15546633	2.806397616
O	12.48148433	12.67187665	3.01290127
O	13.68575476	13.00444234	0.736186712
O	16.03542005	13.06480186	12.92323983

O	18.21739064	13.13349611	1.012758495
O	17.69212074	12.93593935	3.620816102
O	15.15030591	12.86632345	2.933434141
O	13.67206658	14.99778711	2.541548977
O	18.22288946	15.27104305	2.547641996
O	11.53851196	18.82083171	5.176033505
O	11.55471422	16.16006141	5.494338479
O	12.14513744	13.54742095	5.495807752
O	15.74213064	18.83789463	4.816600766
O	16.16369549	16.21620319	5.088029776
O	15.8174941	13.6109474	5.423085826
O	0.125494063	17.09271978	2.815186703
O	20.14383393	13.41231455	2.751555516
O	17.16100046	14.67901402	9.900397036
O	16.27213995	15.21167198	7.498246848
O	13.94632634	15.3471869	6.224724015
O	11.81304739	14.81389898	7.758270737
O	12.12675767	14.93455798	10.39864864
O	14.56590352	14.82485163	9.492142853
O	17.27238864	19.36057457	9.559836848
O	15.95255839	18.62887667	7.428300845
O	13.74049008	19.56350106	6.418933149
O	11.47429126	19.44523127	7.767489319
O	12.08367732	19.36691951	10.3580197
O	14.61841095	19.30050807	9.643347012
O	16.02074425	17.02496683	9.502032158
O	11.62356372	17.13030538	8.960364354
O	18.41417905	13.33467916	11.7785382
O	17.88712617	15.88921305	12.17704699
O	17.81930083	18.56545532	12.04481052
O	13.81270439	13.28851739	11.50403013
O	14.09157487	15.89344718	11.9036607
O	13.9412457	18.57209375	12.11113207
O	9.783683323	15.21249174	9.44775996
O	9.648656146	18.84436556	9.566613189
O	12.46664514	25.59842928	3.142071561
O	13.54586131	25.07513608	0.826519463
O	15.88313818	25.13813675	12.97461955
O	17.88727909	24.66073604	1.239298395
O	17.38044159	25.2720199	3.801480408
O	14.93784136	24.53636689	3.015865021
O	12.80833116	9.252957729	3.487225249
O	13.94379436	9.58373844	1.151563407
O	16.10659693	8.985676981	13.12613372
O	18.27746384	9.182592143	1.2149862
O	17.8866483	9.310591337	3.869930243
O	15.39736425	8.910454206	3.260860215
O	13.74514334	7.15905356	2.086810983
O	18.29103092	6.964878502	2.670534106
O	12.08432578	23.72861041	4.949317768
O	11.67842629	6.023537603	5.678340487
O	11.81474321	8.663117748	5.875840825
O	16.08089014	23.26583356	4.974885932
O	15.2860295	25.76695632	5.361070217

O	16.08852938	8.834714709	5.833872549
O	19.81800348	25.27353637	2.946738316
O	0.225128699	8.727162284	2.878626899
O	17.29179409	7.162009723	10.1703521
O	15.88485147	7.379940185	7.995721481
O	14.0266552	7.169087127	6.080542404
O	12.22034756	7.17049582	8.014582106
O	12.09817655	7.166234892	10.64772185
O	14.67657883	6.775950156	10.23679129
O	17.69609969	23.18394039	9.773521983
O	16.29346901	23.32600693	7.586134752
O	13.94415585	22.78549628	6.564833357
O	11.5375272	23.45732235	7.507585911
O	12.47148193	22.91955332	9.908052997
O	15.09381621	22.58660626	9.845155442
O	15.81452316	25.06659487	9.561789803
O	11.68796252	25.3950414	9.295457912
O	18.49474282	8.705568529	11.98371987
O	18.28976899	25.87063268	12.2349347
O	17.75593969	23.30686745	12.42864293
O	13.75021841	8.451592961	12.12060863
O	13.54444831	5.793571491	12.3855767
O	13.88016469	23.7201662	11.97931902
O	9.920942696	7.52739627	9.303072679
O	9.907482861	23.4575126	9.635680949
O	7.148845911	15.08112191	9.510896863
O	6.218489099	14.76227056	11.98904212
O	3.92499028	15.01294043	-0.062273461
O	1.601133175	14.67655072	12.10260872
O	2.071213471	15.08163861	9.500774113
O	4.599616146	15.36187971	10.03529657
O	7.206143188	19.56669583	10.17085294
O	6.210103822	19.23113208	12.57576725
O	3.796328633	19.51647639	0.131796099
O	1.611130104	19.50745284	12.05349318
O	2.200408297	19.04031847	9.496860874
O	4.62127781	19.16085451	10.43039539
O	6.33991426	17.17991721	10.90452246
O	1.339590916	17.09516357	11.16294916
O	8.489848029	13.74480787	7.691045063
O	8.474801155	16.41401509	7.566956709
O	7.865581781	19.02832768	7.620242753
O	3.734567865	13.61844295	8.174200485
O	3.668940478	16.21827532	7.705297833
O	4.192630189	18.84195146	7.770328821
O	19.6856638	15.14890257	10.36941622
O	19.74871227	19.09790601	10.29815804
O	2.683579443	17.22531564	3.204894121
O	3.93592812	17.50207722	5.463399768
O	6.057330081	17.15359452	7.020003539
O	8.059829139	17.74391618	5.323167247
O	7.677987182	17.15576116	2.740976658
O	5.226202168	16.49985582	3.339494444
O	2.542779527	12.79289784	3.486491705

O	3.939614019	12.67739974	5.682227369
O	6.065105477	12.81007332	7.288493462
O	7.808352778	12.86170096	5.293290668
O	7.69242818	13.46517987	2.745242531
O	5.144739021	13.27272573	3.409191753
O	3.470864256	15.02565989	4.551777429
O	8.540492352	15.27826459	4.480915733
O	1.586751336	18.5752124	1.197826572
O	1.733945448	15.9424554	1.063059979
O	2.073219185	13.33037831	0.925840401
O	5.680289855	18.57928193	1.737805752
O	6.136648262	16.13579163	0.858604389
O	5.868532126	13.4709715	0.857065587
O	10.07541812	17.28707344	3.629425274
O	10.05377734	13.143858	3.887633656
O	8.680434003	21.24659656	8.828903722
O	3.738382862	21.1491454	8.897916652
O	5.739304173	21.099576	1.063175659
O	1.738275425	21.14256218	0.676862683
O	1.195814626	11.0745947	2.01180757
O	6.34164504	11.25585196	2.079409907
O	4.077386112	11.07218429	7.746269318
O	8.157843972	11.1526846	7.294772486
O	11.4758289	11.08861968	4.881922461
O	16.08806204	11.12095488	4.674615475
O	14.41949727	10.99249748	12.4473561
O	17.80938855	11.05224147	12.84292411
O	18.22207602	21.04135286	11.31599668
O	13.68942005	21.10992183	11.44043387
O	15.94420807	21.02745199	6.291556889
O	11.81136548	21.31666574	5.952258277
O	7.152255806	27.28782335	10.18203435
O	6.273130883	27.34792307	12.64887185
O	4.037324413	26.88849891	0.600775626
O	2.012133244	27.48071556	12.38262497
O	2.150174569	27.4160196	9.773332989
O	4.588326406	26.93417212	10.63016078
O	3.80099244	26.01183495	8.253504229
O	19.82681976	27.55175235	10.96702129
O	3.807039386	27.14770038	3.877121075
O	8.143637768	26.87074778	4.614261209
O	1.592663575	26.13338153	1.204424502
O	14.56137357	27.03982879	2.40468201
O	18.09016075	27.12767846	1.998033036
O	11.6865054	26.28161349	5.619106373
O	17.48358017	27.0682525	9.91688142
O	16.22021613	26.86798131	7.624380607
O	13.80281363	27.41810681	6.751921216
O	11.42193401	27.6673558	7.83528732
O	12.22900172	27.7501261	10.37204024
O	14.82114396	27.49024664	9.779329249
O	13.8088393	26.4147137	11.98058232
O	9.739000287	27.14741972	9.776306608
O	2.590007789	2.947145468	9.974730207

O 5.25265914 3.087124313 9.893975668
O 3.878545734 3.480744643 7.674968184
O -0.000974976 3.353538971 10.37614985
O 17.52574877 2.813976508 9.895357897
O 18.22457551 3.471597677 12.38604614
O 7.591335899 29.02461424 3.220208849
O 5.013317753 29.49142704 3.817150804
O 5.737747637 28.87515011 1.327655979
O 17.69757841 29.10394715 3.668974786
O 15.0825542 29.54683618 3.283132965
O 15.95167715 28.34738481 5.486743331
O 6.14288623 3.624863052 1.211139682
O 15.67550395 3.734227686 5.290577188
O 16.14590754 2.745622316 7.699636582
O 4.207233403 28.62478127 8.626853282
O 3.742939679 28.75574149 6.016245773
O 17.56085861 28.39595813 12.16787271
O 6.337774245 2.484731316 12.20272161
O 18.09158131 29.61903308 1.084994551
Om 8.146127554 0.931454201 8.963192091
Sim 8.529298873 2.474795078 8.954429268
Om 1.867667719 1.27395476 0.802580649
Sim 2.395392838 2.712052881 0.334839308
Om 13.5377213 31.43060804 13.1180331
Sim 14.37698135 30.08172596 13.00235308
Hm 13.53263637 0.281079382 11.95448275
Om 13.47535887 0.881355777 11.11452324
Sim 13.68048847 2.469128292 11.05217516
Om 1.198910075 31.08005885 2.142156485
Sim 1.449886593 29.56759602 2.474264525
Om 11.33914947 30.88203901 3.678204748
Sim 11.48606258 29.3416262 4.054325214
Om 11.46860084 1.207119458 6.566938499
Sim 12.43731414 2.485426399 6.505363789
Hm 8.047479124 32.05401758 7.981526917
Om 8.071514071 31.30038968 7.225086921
Sim 7.724473982 29.73967242 7.117789153
Hd 8.033313195 2.07022263 2.292638233
Hd 9.540683253 -0.999341651 0.560584964
Od 7.938869297 1.132556196 2.540840131
Od 8.01803879 -0.40047738 0.271046834
Sid 7.111175531 0.171675836 1.446520747
Hd 21.54160081 32.79395899 13.51570391
Hd 19.04179225 31.27372534 9.279729709
Od 20.97614918 31.9033721 12.17180803
Od 19.82093305 31.36107232 9.861969184
Sid 19.55156969 31.84635443 11.42125244
Hd 1.263616818 -0.399875767 8.617269633
Hd 3.586858267 1.888865557 6.188743233
Od 1.667297438 -0.12800195 7.762378142
Od 3.398300717 0.934056196 6.098874659
Sid 3.253543476 0.161416036 7.546621187
Hd 19.18496422 -0.154236247 6.770094557
Od 18.55410862 0.329314201 4.292340117

Od 18.21883877 -0.270984706 6.831326452
Sid 17.44166984 0.008223393 5.406527879
Or 7.878476919 3.289071951 10.23925639
Or 8.018502335 3.336874039 7.628291144
Or 10.14944084 2.676063853 9.095303368
Or 3.929175589 1.071755176 8.745956321
Or 18.88201597 1.156308806 11.44587578
Or 12.33278283 3.211716564 10.50013467
Or 14.88432084 2.879063071 10.03265956
Or 14.01388703 3.144091414 12.5175318
Or 2.3303081 29.35314474 3.866867362
Or 2.277069952 28.75718061 1.27380903
Or 20.08855126 28.68786926 2.672671651
Or 6.410344113 31.21667883 2.414301033
Or 10.08872196 28.48770113 3.800378879
Or 12.5821249 28.61477439 3.068597147
Or 11.9450814 28.97139719 5.607002074
Or 16.42937412 30.91858439 5.07999531
Or 5.800320702 1.014345411 0.876622811
Or 3.977192212 2.743764929 -0.057567165
Or 2.135209756 3.689281201 1.61238842
Or 16.36044789 1.218300104 5.556730344
Or 13.98082675 2.014052879 6.286984441
Or 11.95820332 3.391047493 5.223884832
Or 12.40048313 3.415759398 7.853500357
Or 4.130863933 30.97189054 7.497761459
Or 6.142757141 29.35964187 6.96153136
Or 8.275624124 28.89160396 8.419533272
Or 8.473562801 29.22894018 5.757506297
Or 18.47511722 30.88532692 12.17785804
Or 16.007907 30.36210787 12.98664382
Or 14.01914909 29.1105806 11.71459318
Or 1.591794203 3.288265406 12.4356767
Or 14.01851793 29.14505496 0.923410099
N3 0.199865833 17.10142079 7.968152516
H3 0.929055625 17.02383146 7.251287646
H3 -0.342621192 17.93829565 7.728697017
H3 -0.425445916 16.30256946 7.816566293
N3 4.017971063 9.779920538 12.18031121
H3 4.592579738 9.833496974 11.33172527
H3 4.450890582 10.43875449 12.83722003
H3 3.101042987 10.19261156 11.92454568
N3 10.52492192 17.04123133 -1.159758429
H3 9.659138529 17.58782762 -1.214017653
H3 10.9387546 17.26617675 -0.248229815
H3 10.23271863 16.05980027 -1.099417192
N3 10.47192917 30.6918508 0.801918616
H3 10.38979059 29.63280281 0.624471265
H3 10.68250923 30.82329679 1.816458214
H3 11.26549706 31.05791183 0.248895608
N3 10.27555476 27.88473781 13.84137527
H3 10.57872637 27.46783071 14.72988689
H3 10.86124159 27.47344555 13.10592766
H3 9.321473383 27.5462841 13.67250885

N3	16.14841467	10.32666841	9.894216002
H3	16.24329599	9.783194505	9.028871242
H3	16.8935145	11.03147711	9.856414251
H3	15.27051792	10.8492916	9.799820928
N3	1.266152968	10.99865986	11.24166867
H3	1.33814811	11.08953989	10.22226319
H3	1.003084071	11.92807855	11.58735795
H3	0.454452586	10.39489962	11.41399075
N3	7.199814895	-0.340262859	11.18783454
H3	6.262542166	0.074186031	11.2535566
H3	7.099285564	-1.317748812	10.89192078
H3	7.702376385	0.197432952	10.40563405
Hm	7.630942787	-0.315511686	12.22862326
N3	18.69958907	0.538267122	1.738548496
H3	17.93567264	0.234846188	1.128851301
H3	18.4659096	0.432833351	3.230158217
H3	19.49933855	-0.10375255	1.585179111
Hd	18.98642252	1.464741458	1.405410667
N3	11.32588188	31.92656436	8.688004811
H3	10.37921214	32.12745371	9.037457391
H3	11.99420839	32.15623984	9.437839134
H3	11.39044941	30.91946321	8.50700168
Hm	11.4622498	32.75032498	7.456707734
N3	13.37391638	-0.051361032	2.365096091
H3	13.41783294	-0.301935337	1.300300864
H3	12.55216695	-0.522556706	2.897059514
H3	14.24258442	-0.375827847	2.81281157
Hm	13.31720051	0.967905512	2.476653259
N3	3.063456634	30.43483008	11.84042192
H3	2.828933518	29.4365441	11.88771077
H3	3.655509611	30.63968882	12.65307526
H3	3.612404945	30.58294918	10.98777877
Hm	1.770832562	31.26113208	11.97077138
N3	1.403717965	0.783158465	3.794919033
H3	2.117131968	0.711300453	4.536915586
H3	1.571691893	1.634013395	3.247685478
H3	1.390410073	-0.138134922	3.029627142
Hm	0.460157588	0.82516158	4.218661431
N3	9.470838788	32.42630868	4.93571145
H3	10.13855726	31.74825943	4.450219814
H3	10.09466975	33.07576	5.469689165
H3	8.881252457	32.89428016	4.223492226
Hm	8.877647159	31.94390129	5.64395507

REFERENCES

Allen, M. P.; Tildesley, D. J. *Computer Simulations of Liquids*; Oxford Science Publications, 1994.
